# Supplementary material for: Plasma exchange and radiation resensitize immunotherapy-refractory melanoma: a phase I trial
Source: Nat Commun. 2025 Mar 13;16:2507. doi: 10.1038/s41467-025-57865-9 (PMC11906856; doi:10.1038/s41467-025-57865-9)
Supplement: Supplementary file 1 — Supplementary Information [file 41467_2025_57865_MOESM1_ESM.pdf]

Nature Communications: Supplementary Information

Title: Plasma exchange and radiation resensitize immunotherapy-refractory melanoma: a phase I trial.

Supplementary Figures

Supplementary Figure 1

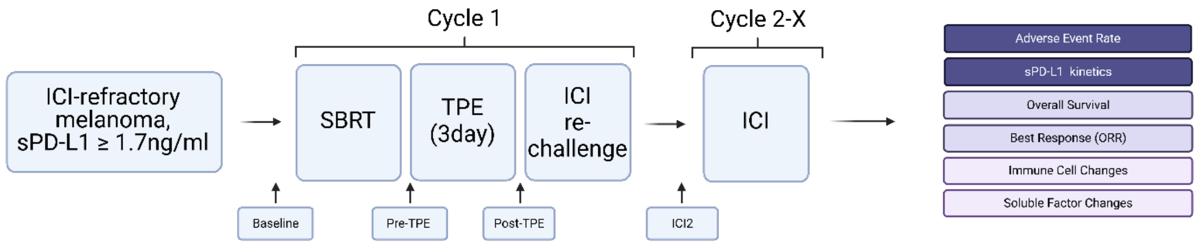

Supplementary Figure 1 – Treatment schema. Eligible patients with ICI-refractory melanoma and sPD-L1 ≥1.7 ng/ml received radiation followed by three sessions of therapeutic plasma exchange (once daily) followed by re-challenge with ICI. Blood samples were taken at registration before SBRT (baseline), after SBRT and before TPE (pre-TPE), after TPE and before ICI re-challenge (post-TPE), and before the second cycle of ICI re-challenge (ICI2). SBRT = stereotactic body radiotherapy, TPE = therapeutic plasma exchange. ICI = Immune checkpoint inhibitor therapy. Created in BioRender. Orme, J. (2025) https://BioRender.com/q80l745.

Supplementary Figure 2

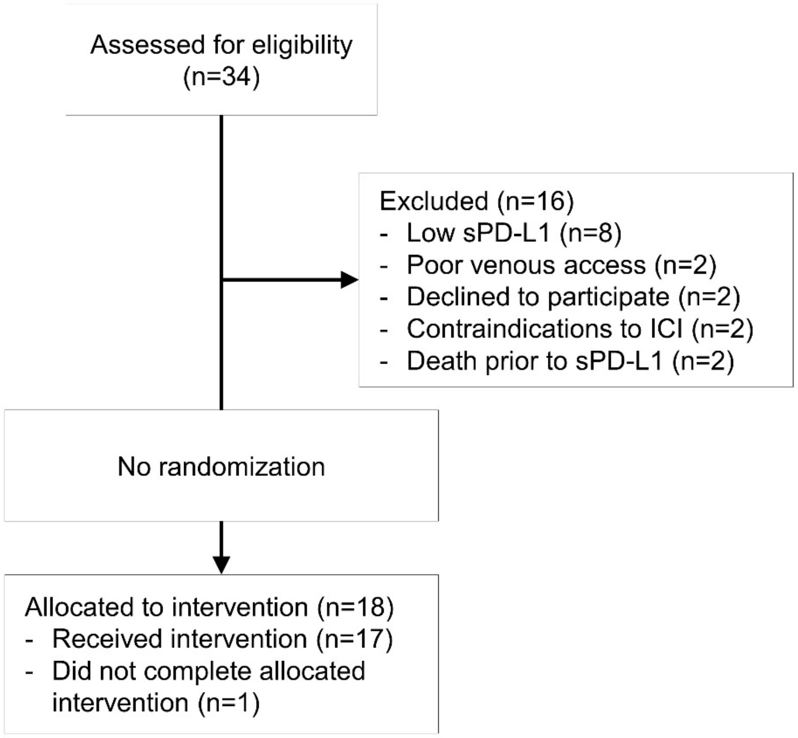

Supplementary Figure 2 – **CONSORT diagram.** 34 patients were assessed for eligibility and 18 were eligible for intervention. 16 were excluded due to exclusion criteria as listed. 17 of 18 patients who were determined to be eligible completed the intervention (one patient completed only two of three TPE sessions due to grade 4 sepsis but did undergo ICI re-challenge).

Supplementary Figure 3

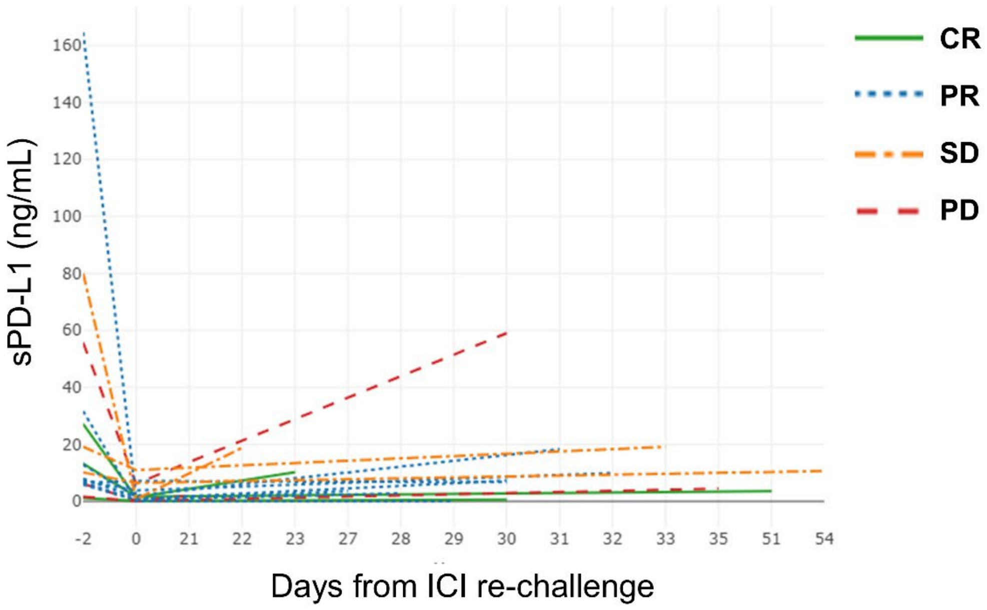

Supplementary Figure 3 – Absolute changes in sPD-L1. Absolute sPD-L1 levels (in ng/mL) by patient over the course of treatment from day of ICI re-challenge. **See Fig 1A**. CR (green solid): complete response, defined as absence of radiologically apparent disease. PR (blue dotted): partial response, defined as greater than 30% radiographic reduction. SD (orange dot-dashed): stable disease, defined as less than 30% radiographic reduction. PD (red dashed): progressing disease, defined as increase of any lesion by 20%. N=18.

Supplementary Figure 4

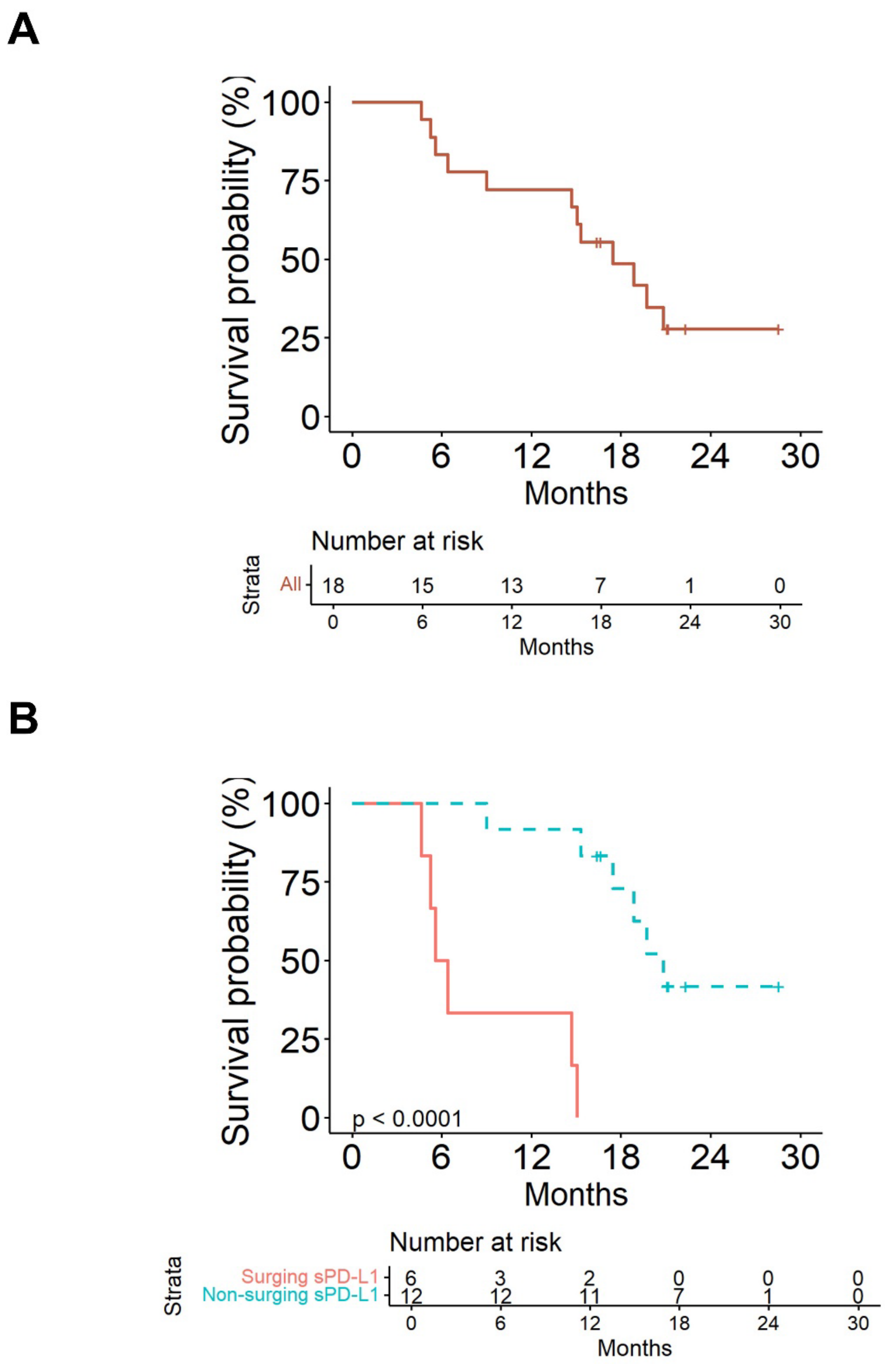

Supplementary Figure 4. **Overall survival for the cohort**. **(A)** Median OS was 17.4 months (95% CI 14.7-NR) by the Kaplan-Meier method. **(B)** Patients with high re-

accumulation of sPD-L1 experienced inferior OS (log rank p<0.0001, Cox proportional HR 27.7 [95% CI 3-240], p=0.003).

Supplementary Figure 5

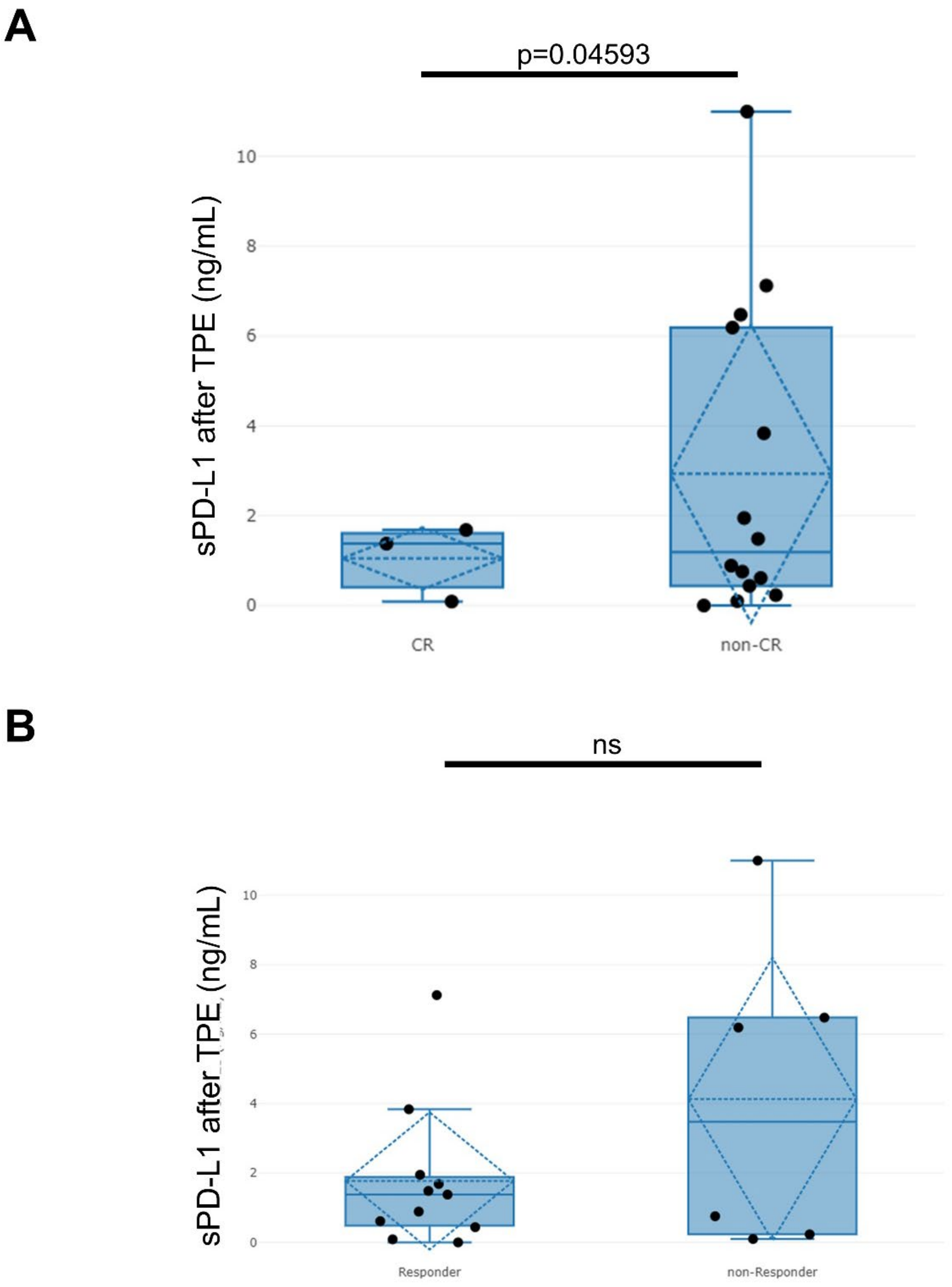

Supplementary Figure 5 – **Response and outcomes**. **(A)** Patients experiencing CR (n=3) had lower absolute sPDL1 levels (mean 1.05 ng/mL) than those without CR (n=15, mean 2.9ng/ml) after TPE (Student's t test p=0.046). **(B)** Post-TPE sPD-L1 level

comparisons for responders (n=12, mean 1.77 ng/ml) versus non-responders (n=6, mean 4.12 ng/ml) were not statistically significant (Student's t test p=0.13).

Supplementary Figure 6

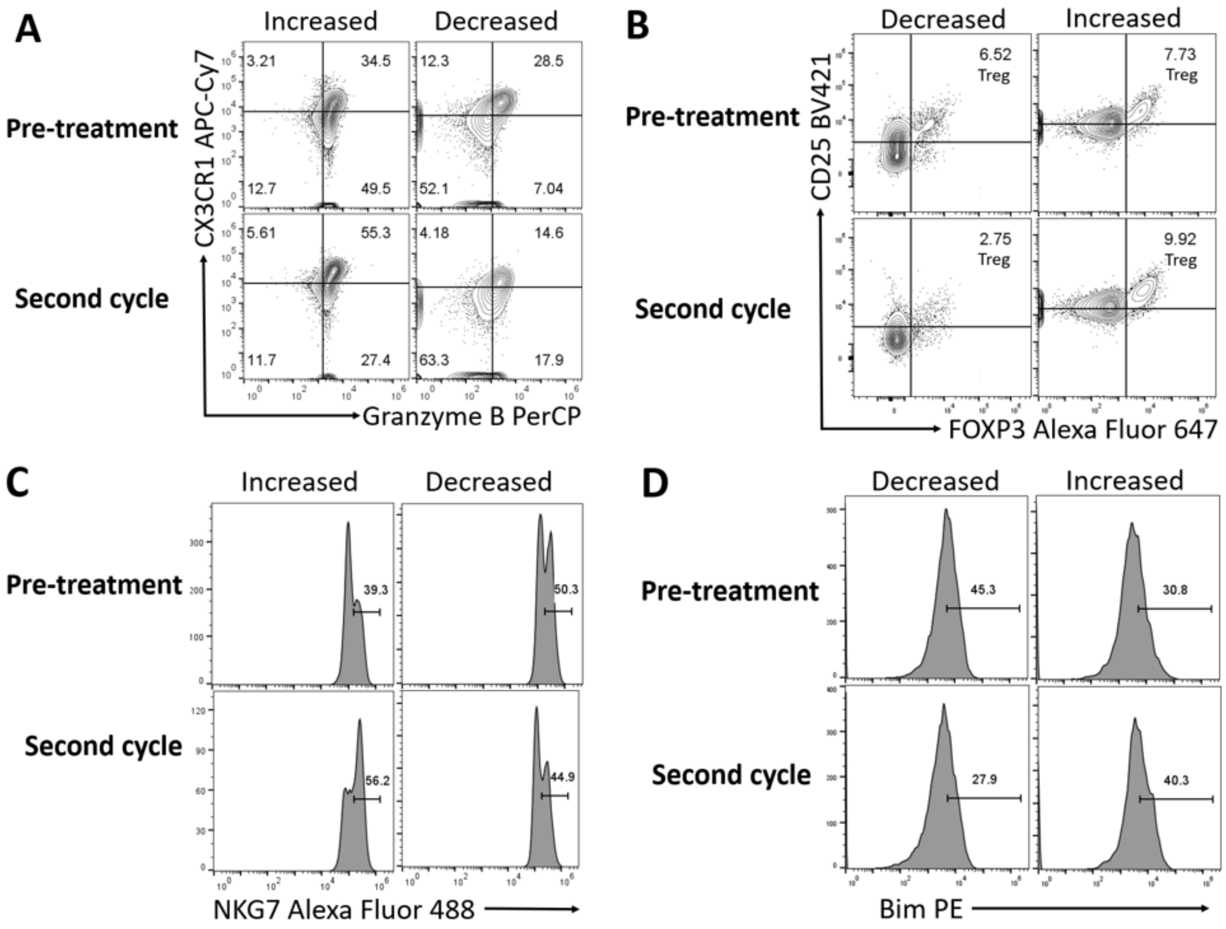

Supplementary Figure 6 – Representative flow cytometry plots. Representative flow cytometry plots showing the changes of peripheral immune cell populations from pre-treatment to the second cycle of ICI re-challenge.

Supplementary Figure 7

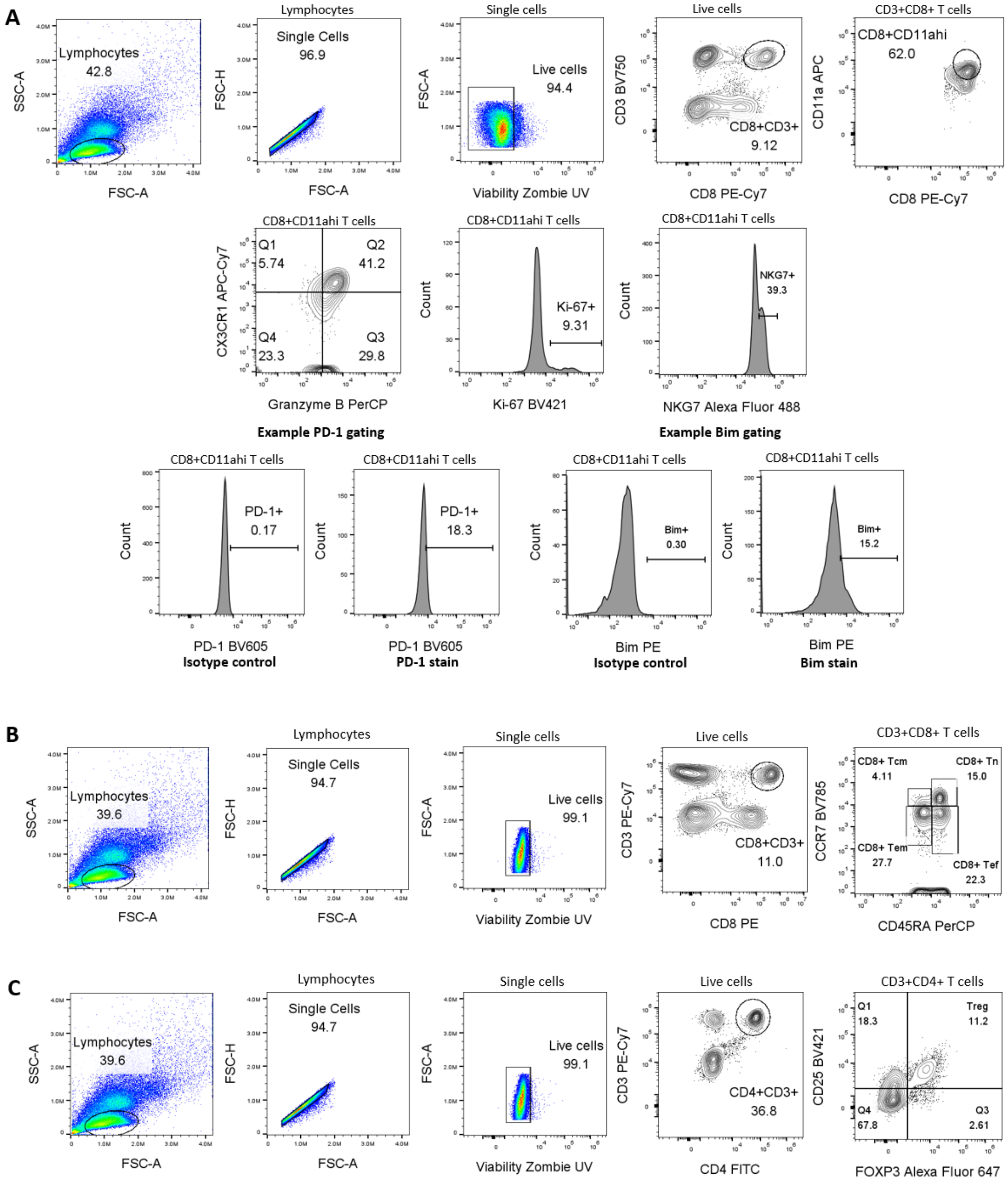

D

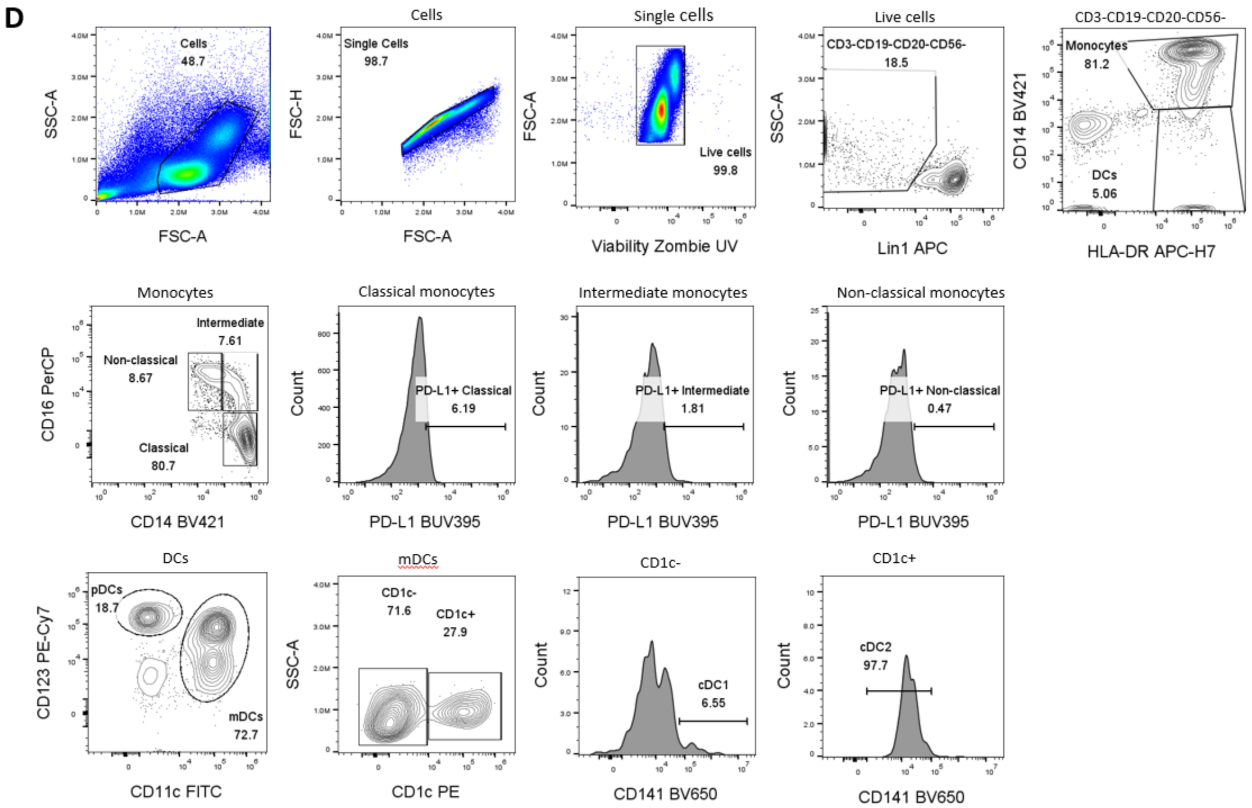

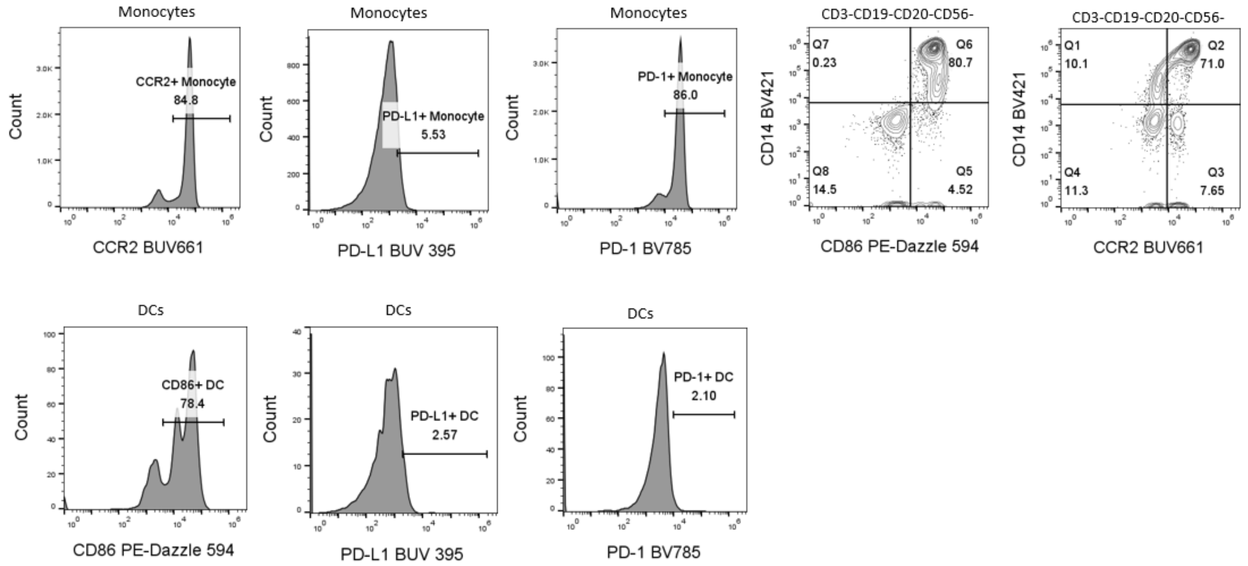

Supplementary Figure 7 – Flow cytometry gating strategies. **(A)** Flow cytometry gating strategies of tumor reactive ($CD8^+CD11a^{high}$) T cell population analysis. **(B)** Flow cytometry gating strategies of central memory (T_{CM}), effector memory (T_{EM}), effector

(T_{EF}), and naïve (T_N) T-cells populations. **(C)** Flow cytometry gating strategies of regulatory T cells (Treg). **(D)** Flow cytometry gating strategies of monocytes and dendritic cells (DCs).

Supplementary Figure 8

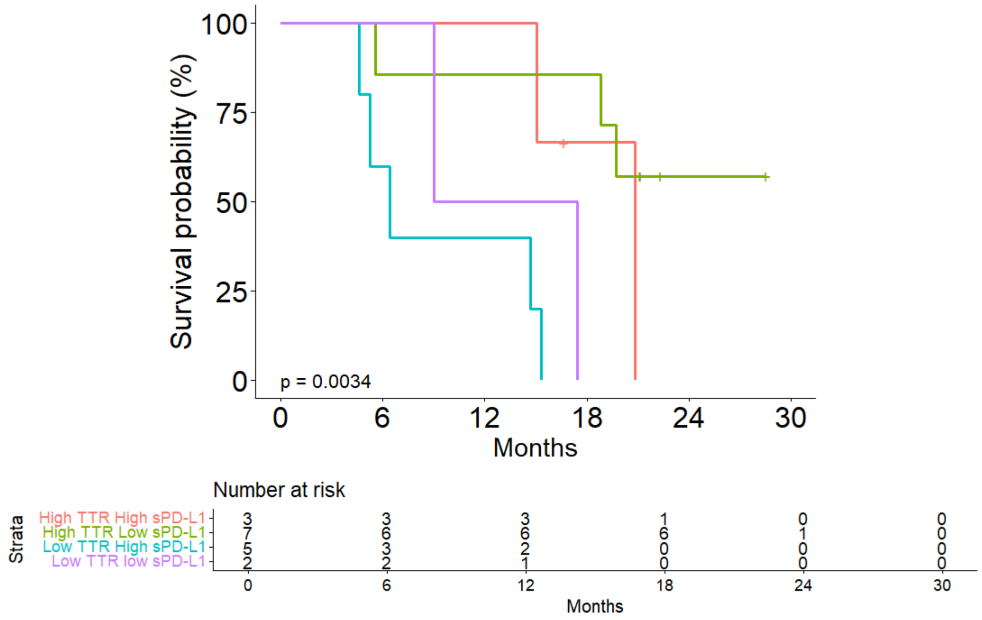

Supplementary Figure 8 – A combination of low post-TPE sPD-L1 and increasing T_{TR} predicts superior overall survival. A combination of low post-TPE sPD-L1 and increasing T_{TR} predicts superior overall survival (composite log rank p=0.0034). Cox proportional hazard ratio for each group versus low T_{TR} and high sPD-L1 were 0.49 (low T_{TR} and low sPD-L1, 95% CI [confidence interval] 0.08-2.87, p=0.425), 0.23 (high T_{TR} and high sPD-L1, 95% CI 0.04-1.23, p=0.085), and 0.07 (high T_{TR} and low sPD-L1, 95% CI 0.01-0.42, p=0.004). All statistical tests were two-sided.

Supplementary Figure 9

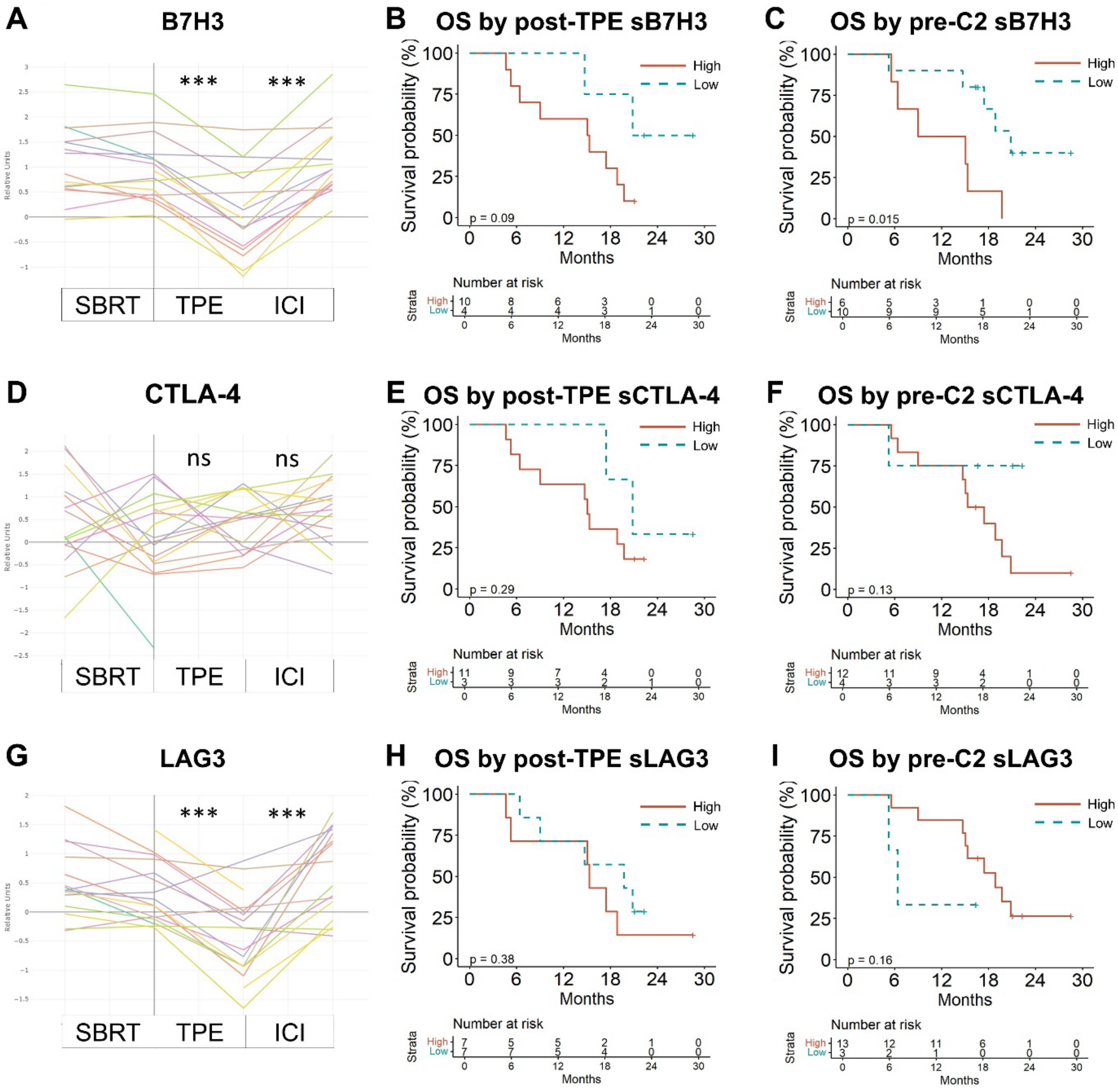

Supplementary Figure 9 – TPE removes soluble factors and post-TPE or ICI2 levels may correlate with outcomes. (A) TPE significantly decreased circulating B7H3 (Wilcoxon p=0.0001), with significant recovery before ICI2 (Wilcoxon p=0.0001). **(B)** OS is estimated in patients high versus low B7H3 after TPE. Patients with high B7H3 experienced inferior OS (log rank p=0.09, HR 0.27 [95% CI 0.06-0.1.33]). **(C)** OS is estimated in patients high versus low B7H3 before cycle 2 of ICI re-challenge.

(D) TPE did not decrease circulating CTLA-4 (Wilcoxon p=0.73), nor did CTLA-4 rise significantly before ICI2 (Wilcoxon p=0.25). **(E)** OS is estimated in patients high versus low CTLA-4 after TPE, and a statisticall significant difference was not found (log rank p=0.29, HR 0.44 [95% CI 0.09-2.07]). **(F)** OS is estimated in patients high versus low CTLA-4 before cycle 2 of ICI re-challenge, and a statisticall significant difference was not found (log rank p=0.13, HR 0.23 [95% CI 0.03-1.83]). **(G)** TPE significantly decreased circulating LAG3 (Wilcoxon p=0.0001), with significant recovery before ICI2 (Wilcoxon p=0.0004). **(H)** OS is estimated in patients high versus low LAG3 after TPE, and a statisticall significant difference was not found (log rank p=0.38, HR 0.58 [95% CI 0.17-1.97]). **(I)** OS is estimated in patients high versus low LAG3 before cycle 2 of ICI re-challenge, and a statisticall significant difference was not found (log rank p=0.16, HR 3.1 [95% CI 0.559-16.33]). All statistical tests were two-sided, no adjustments were made for multiple comparisons. **See also Fig 3.**

Supplementary Tables

Table S1

SBRT		
Lesions per patient[1]		
	Irradiated	1.5 (1-4) lesions
	Non-irradiated	9.5 (3-32) lesions
	Percent irradiated	12% (6-33%)
Radiation parameters[1]	Total radiation dose per lesion	40 Gy (25-60 Gy)
	Radiation fractions per lesion	5 (3-10)
Radiated lesion location[2]		
	Visceral only	12/18 (66.7%)
	Bone only	2/18 (11.1%)
	Mixed visceral and bone	3/18 (16.7%)
	Brain	1/18 (5.6%)

[1] Summarized as median (range)
[2] Summarized as n/N (%)

Supplementary Table 1. Characteristics of lesions and radiation in patients on the study. For additional patient-level information, please see **Data File 1**.

Table S2

Cell Subtypes	Markers
Treg	CD3+ CD4+ CD25+ FOXP3+
CD8+ T central memory	CD3+ CD8+ CCR7+ CD45RA-
CD8+ T effector	CD3+ CD8+ CCR7- CD45RA+
CD8+ T effector memory	CD3+ CD8+ CCR7- CD45RA-
CD8+ T naive	CD3+ CD8+ CCR7+ CD45RA+
CD8+CD11ahi	CD3+ CD8+ CD11ahi
Bim+/CD8+CD11ahi	CD3+ CD8+ CD11ahi Bim+
Ki-67+/CD8+CD11ahi	CD3+ CD8+ CD11ahi Ki-67+
NKG7+/CD8+CD11ahi	CD3+ CD8+ CD11ahi NKG7+
PD-1+/CD8+CD11ahi	CD3+ CD8+ CD11ahi PD-1+
Granzyme B+ CX3CR1+/CD8+CD11ahi	CD3+ CD8+ CD11ahi Granzyme B+ CX3CR1+
Monocytes	CD3- CD19- CD20- CD56- CD14+ HLA-DR+
DC	CD3- CD19- CD20- CD56- CD14- HLA-DR+

Non-classical monocyte	CD3- CD19- CD20- CD56- HLA-DR+ CD14+ CD16++
Classical monocyte	CD3- CD19- CD20- CD56- HLA-DR+ CD14++ CD16-
Intermediate monocyte	CD3- CD19- CD20- CD56- HLA-DR+ CD14++ CD16+
pDCs	CD3- CD19- CD20- CD56- HLA-DR+ CD14- CD123+ CD11c-
mDCs	CD3- CD19- CD20- CD56- HLA-DR+ CD14- CD123- CD11c+
cDC1	CD3- CD19- CD20- CD56- HLA-DR+ CD14- CD123- CD11c+ CD141+ CD1c-
cDC2	CD3- CD19- CD20- CD56- HLA-DR+ CD14- CD123- CD11c+ CD141- CD1c+
PD-L1+ Monocyte	CD3- CD19- CD20- CD56- CD14+ HLA-DR+ PD-L1+
PD-L1+ Classical monocyte	CD3- CD19- CD20- CD56- HLA-DR+ CD14++ CD16- PD-L1+
PD-L1+ Intermediate monocyte	CD3- CD19- CD20- CD56- HLA-DR+ CD14++ CD16+ PD-L1+
PD-L1+ Non-classical monocyte	CD3- CD19- CD20- CD56- HLA-DR+ CD14+ CD16++ PD-L1+
PD-1+ Monocyte	CD3- CD19- CD20- CD56- CD14+ HLA-DR+ PD-1+
PD-1+ DC	CD3- CD19- CD20- CD56- CD14- HLA-DR+ PD-1+
PD-L1+DC	CD3- CD19- CD20- CD56- CD14- HLA-DR+ PD-L1+

CCR2+ Monocyte	CD3- CD19- CD20- CD56- CD14+ HLA-DR+ CCR2+
CCR2+ CD14+	CD3- CD19- CD20- CD56- CD14+ CCR2+
CD14+ CD86+	CD3- CD19- CD20- CD56- CD14+ CD86+
CD86+ DC	CD3- CD19- CD20- CD56- CD14- HLA-DR+ CD86+

Supplementary Table 2. Markers used to determine identity of peripheral blood mononuclear cells (PBMC) on flow cytometry.

Table S3

Antibody	Vendor	Clone	Catalog no.
CD3 BV750	Biolegend	SK7	344846
CD8 PE/Cy7	BD Pharmingen	RPA-T8	557746
CD11a APC	Biolegend	HI111	301212
PD-1 BV605	Biolegend	EH12.2H7	329924
CX3CR1 APC/Cy7	Biolegend	2A9-1	341616
Granzyme B Percp	Novus Biological	CLB-GB11	NBP-1-50071PCP
Bim PE	Cell Signaling	C34C5	12186S
Ki-67 BV421	BD Horizon	B56	562899
NKG7	In-house, Fusion Antibodies	8H3/8K3	NA
CD3 PE/Cy7	Biolegend	HIT3a	300316
CD4 FITC	Biolegend	SK3	344604
CD25 BV421	Biolegend	2A3	302630
CD8 PE	Biolegend	SK1	344705
CD45RA PerCP	Biolegend	HI100	304156
CCR7 BV785	Biolegend	G043H7	353230
FoxP3 Alexa Fluor 647	BD Pharmingen	236A/E7	561184
Lin1(CD3, CD19, CD20, CD56) APC	Biolegend	UCHT1; HIB19; 2H7; 5.1H11;	363601
CD14 BV421	BD Horizon	MφP9	563743
CD16 PerCP	Biolegend	3G8	302030
HLA-DR APC-H7	BD Pharmingen	G46-6	561358
CD11c FITC	BD Pharmingen	B-ly6	561355
CD123 PE-Cy	BD Pharmingen	7G3	560826
CD1c PE	BD Pharmingen	F10/21A3	564900
CD141 BV650	BD Horizon	1A4	569392
CCR2 BUV661	BD OptiBuild	LS132.1D9	750472
CD86 PE/Dazzle™ 594	Biolegend	BU63	374218
PD-1 BV785	Biolegend	EH12.2H7	329930
PD-L1 BUV395	BD OptiBuild	MIH1	740320

Supplementary Table 3. Antibodies used to determine identity of peripheral blood mononuclear cells (PBMC) on flow cytometry.

Table S4

**Population**	**HR**	**Cutoff**	**n_above_cutoff**	**P**	**Hochberg**
Treg	9.72 (95% CI 2-47.32)	1.0255475	9	0.005	*Prespec*
CD8+Tcm	0.55 (95% CI 0.17-1.71)	0.8220340	11	0.299	
CD8+Tef	0.15 (95% CI 0.03-0.72)	0.9338522	9	0.018	0.07
CD8+Tem	0.16 (95% CI 0.02-1.22)	1.1263384	5	0.076	
CD8+Tn	6.03 (95% CI 1.23-29.49)	0.8314608	10	0.027	0.11
CD8+CD11ahi	0.4 (95% CI 0.12-1.35)	0.9806531	9	0.141	
Bim+/CD8+CD11ahi	5.45 (95% CI 1.42-20.95)	0.9903383	5	0.014	*Prespec*
Ki-67+/CD8+CD11ahi	0.39 (95% CI 0.12-1.25)	0.9259260	12	0.112	
NKG7+/CD8+CD11ahi	0.32 (95% CI 0.1-1.07)	0.8913581	12	0.064	
PD-1+/CD8+CD11ahi	0.18 (95% CI 0.05-0.64)	0.7263159	11	0.008	0.032
GzmB+CX3CR1+CD8+CD11ahi	0.15 (95% CI 0.04-0.6)	0.7918315	12	0.008	0.032
Monocytes	4.77 (95% CI 1.35-16.83)	1.1140778	5	0.015	
Dendritic Cells	0.14 (95% CI 0.04-0.53)	0.7887565	9	0.004	0.02
Non-classical monocyte	0.08 (95% CI 0.02-0.39)	0.3729168	11	0.002	0.01
Classical monocyte	3.2 (95% CI 0.88-11.63)	1.0330297	7	0.077	
Intermediate monocyte	0.66 (95% CI 0.21-2.1)	0.9583334	8	0.483	
pDCs	3.58 (95% CI 1.08-11.89)	1.1965813	5	0.037	0.15
mDCs	0.31 (95% CI 0.08-1.15)	0.9811067	8	0.079	
cDC1	2.99 (95% CI 0.87-10.26)	1.7485300	5	0.082	
cDC2	3.98 (95% CI 1.25-12.72)	1.4827534	7	0.020	0.08
PD-L1+ Monocyte	0.5 (95% CI 0.16-1.59)	0.3780489	12	0.241	
PD-L1+ Classical monocyte	0.44 (95% CI 0.13-1.47)	0.3065328	13	0.182	
PD-L1+ Intermediate monocyte	0.32 (95% CI 0.1-1)	0.3341015	12	0.049	
PD-L1+ Non-classical monocyte	0.42 (95% CI 0.13-1.41)	0.2809525	8	0.160	
PD-1+ Monocyte	4.33 (95% CI 0.56-33.66)	0.9439463	14	0.162	
PD-1+ DC	0.31 (95% CI 0.1-0.99)	0.8671330	11	0.048	0.19

Population	HR	Cutoff	n_above_cutoff	P	Hochberg
PD-L1+DC	2.11 (95% CI 0.66-6.72)	2.1826088	6	0.208	
CCR2+ Monocyte	3.15 (95% CI 0.95-10.44)	90.3000001	6	0.060	
CCR2+ CD14+	2.81 (95% CI 0.8-9.86)	81.4000001	8	0.108	
CD14+ CD86+	7.08 (95% CI 0.9-55.69)	83.3000001	13	0.063	
CD86+ DC	1.73 (95% CI 0.52-5.81)	58.1000001	11	0.375	

Supplementary Table 4. Hazard ratios and 95% confidence intervals for death by PBMC subpopulation change from baseline to second cycle of ICI re-challenge. Cox proportional hazards modeling was performed at each predicted cutoff. All statistical tests were two-sided. Hochberg adjustment for multiple comparisons is shown where appropriate. See **Table S5** for uniform cutoff of 1.

Table S5

Population	HR	Cutoff	n_above_cutoff	p	Hochberg
Treg	3.69 (95% CI 0.95-14.34)	1	11	0.059	*Prespec*
CD8+Tcm	0.88 (95% CI 0.26-2.99)	1	6	0.838	
CD8+Tef	0.56 (95% CI 0.16-1.89)	1	7	0.347	
CD8+Tem	0.64 (95% CI 0.19-2.12)	1	8	0.461	
CD8+Tn	3.02 (95% CI 0.83-11)	1	7	0.094	
CD8+CD11ahi	0.41 (95% CI 0.11-1.53)	1	7	0.185	
Bim+/CD8+CD11ahi	5.45 (95% CI 1.42-20.95)	1	5	0.014	*Prespec*
Ki-67+/CD8+CD11ahi	0.5 (95% CI 0.15-1.62)	1	11	0.247	
NKG7+/CD8+CD11ahi	0.56 (95% CI 0.17-1.89)	1	7	0.349	
PD-1+/CD8+CD11ahi	0.3 (95% CI 0.09-1.01)	1	9	0.051	
GzmB+CX3CR1+CD8+CD11ahi	0.12 (95% CI 0.02-0.62)	1	10	0.011	0.044
Monocytes	4.33 (95% CI 0.56-33.66)	1	14	0.162	
Dendritic Cells	0.14 (95% CI 0.04-0.53)	1	9	0.004	0.02
Non-classical monocyte	0.42 (95% CI 0.09-1.96)	1	4	0.271	
Classical monocyte	1.5 (95% CI 0.4-5.61)	1	13	0.549	
Intermediate monocyte	0.66 (95% CI 0.21-2.1)	1	8	0.483	
pDCs	1.67 (95% CI 0.53-5.2)	1	7	0.379	
mDCs	0.57 (95% CI 0.15-2.1)	1	6	0.396	
cDC1	1.53 (95% CI 0.45-5.2)	1	6	0.497	
cDC2	1.83 (95% CI 0.55-6.13)	1	10	0.325	
PD-L1+ Monocyte	1.2 (95% CI 0.38-3.74)	1	8	0.753	
PD-L1+ Classical monocyte	1.2 (95% CI 0.38-3.74)	1	8	0.753	
PD-L1+ Intermediate monocyte	0.79 (95% CI 0.25-2.53)	1	10	0.696	
PD-L1+ Non-classical monocyte	0.63 (95% CI 0.19-2.11)	1	7	0.457	
PD-1+ Monocyte	2.59 (95% CI 0.69-9.66)	1	11	0.157	
PD-1+ DC	0.38 (95% CI 0.12-1.23)	1	10	0.106	

Population	HR	Cutoff	n_above_cutoff	p	Hochberg
PD-L1+DC	1.26 (95% CI 0.4-4.01)	1	10	0.691	
CCR2+ Monocyte	Inf (95% CI Inf-Inf)	1	11	1	
CCR2+ CD14+	Inf (95% CI Inf-Inf)	1	11	1	
CD14+ CD86+	Inf (95% CI Inf-Inf)	1	11	1	
CD86+ DC	Inf (95% CI Inf-Inf)	1	11	1	

Supplementary Table 5. Hazard ratios and 95% confidence intervals for death by PBMC subpopulation increase from baseline to second cycle of ICI re-challenge. Cox proportional hazards modeling was performed comparing patients with increasing (ratio greater than 1) versus decreasing (ratio less than 1). All statistical tests were two-sided. Hochberg adjustment for multiple comparisons is shown where appropriate. See **Table S4** for variable cutoff.

MC200703: Radiation Therapy, Plasma Exchange, and Immunotherapy in Melanoma

Study Chairs: Jacob Orme, M.D., Ph.D.
Mayo Clinic
Department of Radiation Oncology
200 First Street SW
Rochester MN 55905

Study Co-chairs: Sean Park, M.D., Ph.D., MS
Jeffrey Winters, M.D.
Haidong Dong, M.D., Ph.D.
Fabrice Lucien, Ph.D.
Svetomir Markovic, M.D., Ph.D.
Matthew Block, M.D., Ph.D.
Robert McWilliams, M.D.
Lisa Kottschade, APRN, C.N.P.

Statistician: Nathan Foster, MS √

Study Contributor(s): Dawn M. Deno, BS, CCRP √

√ Study contributor(s) not responsible for patient care

Document History

Version 1.0	4/2/2020
Version 2.0	10/14/2020
Version 3.0	2/8/2021
Version 4.0	11/11/2021
Version 5.0	12/15/2021
Version 6.0	12/9/2022

Protocol Resources

Questions:	Contact Name:
Patient eligibility*, test schedule, treatment delays/interruptions/adjustments, dose modifications, adverse events, forms completion and submission, adverse events, forms completion and submission	Radiation Oncology Study Team Contact email listed in the consent form
Protocol document, consent form, Regulatory issues	Dawn M. Deno, BS, CCRP<br>Phone: 507-293-7964<br>E-mail: deno.dawn@mayo.edu
Statistician	Nathan Foster, MS<br>Phone: 507-284-5051<br>E-mail: foster.nathan@mayo.edu

Table of Contents

Protocol Resources 2
Table of Contents 3
Study Schema 4
List of Abbreviations 5
1. Background 6
2. Goals 8
3. Patient Eligibility 9
4. Test Schedule 10
5. Grouping Factors 13
6. Registration Procedures 14
7. Protocol Treatment 15
8. Dosage Modification Based on Adverse Events 17
9. Ancillary Treatment/Supportive Care 18
10. Adverse Event (AE) Monitoring and Reporting 19
11. Treatment Evaluation/Measurement of Effect 25
12. Descriptive Factors 33
13. Treatment/Follow–up Decision at Evaluation of Patient 34
14. Body Fluid Biospecimens 35
15. Drug Information 37
16. Statistical Considerations and Methodology 39
17. Pathology Considerations/Tissue Biospecimens 43
18. Records and Data Collection Procedures 44
19. Budget 46
20. References 47
Appendix I ECOG Performance Status 49
Appendix II - Immune-Related Adverse Events Questionnaire .... **Error! Bookmark not defined.**

Study Schema

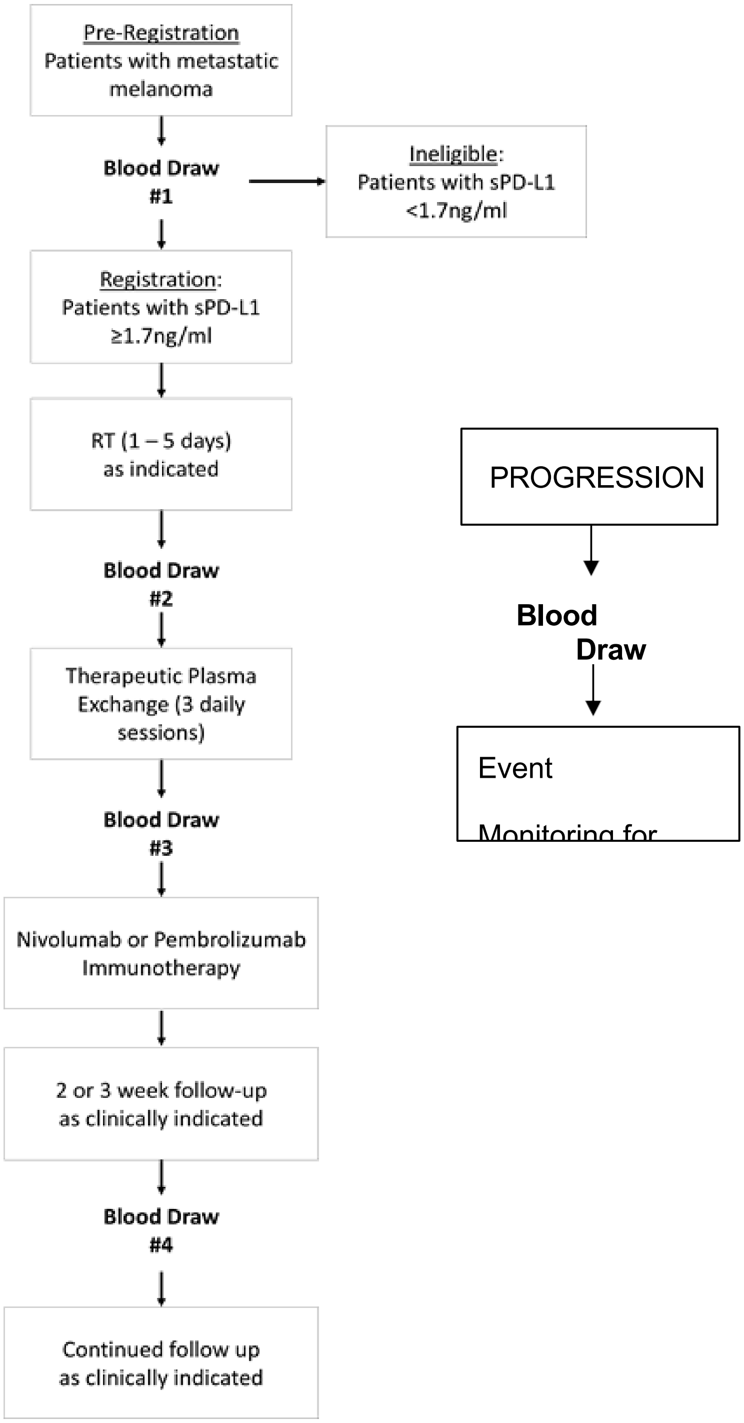

List of Abbreviations

AE	Adverse Event/Adverse Experience
CFR	Code of Federal Regulations
CRF	Case Report Form
DSMB	Data and Safety Monitoring Board
FDA	Food and Drug Administration
GCP	Good Clinical Practice
HIPAA	Health Insurance Portability and Accountability Act
PBT	Proton beam therapy
PI	Principal Investigator
PHI	Protected Health Information
SAE	Serious Adverse Event/Serious Adverse Experience
IRB	Institutional Review Board
ReRT	Re-irradiation
OS	Overall survival
PFS	Progression-free survival
LRC	Locoregional control
RTOG	Radiation Therapy Oncology Group
ECOG	Eastern Cooperative Oncology Group
SFO	Single Field Optimization
MFO	Multi Field Optimization
IFSO	Individual Field Simultaneous Optimization
IMPT	Intensity Modulated Proton Therapy
SPB	Scanning Proton Beam
GTV	Gross Tumor Volume
CTV	Clinical Tumor Volume
OTV	Optimization Target Volume
STV	Scanning Target Volume
PRV	Planning Risk Volume
OAR	Organs at Risk
RU	Range Uncertainty
PET	Positron Emission Tomography
QoL	Quality of Life
EDC	Electronic Data Collection
LET	Linear Energy Transfer
SOC	System Organ Class
RECIST	Response Evaluation Criteria in Solid Tumors
ULN	Upper limit of normal
ORR	Objective Response Rate

1. Background

1.1 Immunotherapy resistance

Almost 3 million patients will receive anti-PD-1 or anti-PD-L1 (henceforth "PD-(L)1 inhibitor") immune checkpoint inhibitors (ICI) to treat their cancer [1], and yet merely 1 in 8 will respond to the treatment [2]. Furthermore, despite initial response to ICI, a majority of the patients succumb to their cancer due to the development of ICI resistance [3]. Recently, we and others have discovered extracellular forms of PD-L1 that mediate PD-(L)1 inhibitor resistance (Fig 1). First, tumors transcribe and secrete soluble PD-L1 (sPD-L1) splice variants [4], [5], and enzymes ADAM10 and ADAM17 also shed sPD-L1 ectodomain directly from the tumor cell surface [6], [7]. Second, tumors generate extracellular vesicles (EVs) bearing surface PD-L1 (evPD-L1) [8], [9]. These extracellular forms of PD-L1 outcompete PD-(L)1 inhibitors, engage and kill CD8+ T cells, and suppress anti-tumor immunity (Fig 1). These forms of PD-L1 at high levels in patient plasma predict poor outcomes in many malignancies [4], [10]–[15].

Thus, *__there is a critical need to develop methods to inhibit or remove extracellular PD-L1__*. In the absence of such discoveries, most patients who do not respond to PD- (L)1 inhibitors will continue to experience poor survival.

Fig 1 – Extracellular forms of PD-L1 (sPD-L1 and evPD-L1) cause resistance to PD-(L)1 inhibitor therapy in cancer by outcompeting PD-(L)1 inhibitors, killing CD8+ T cells, and limiting anti-tumor immunity.

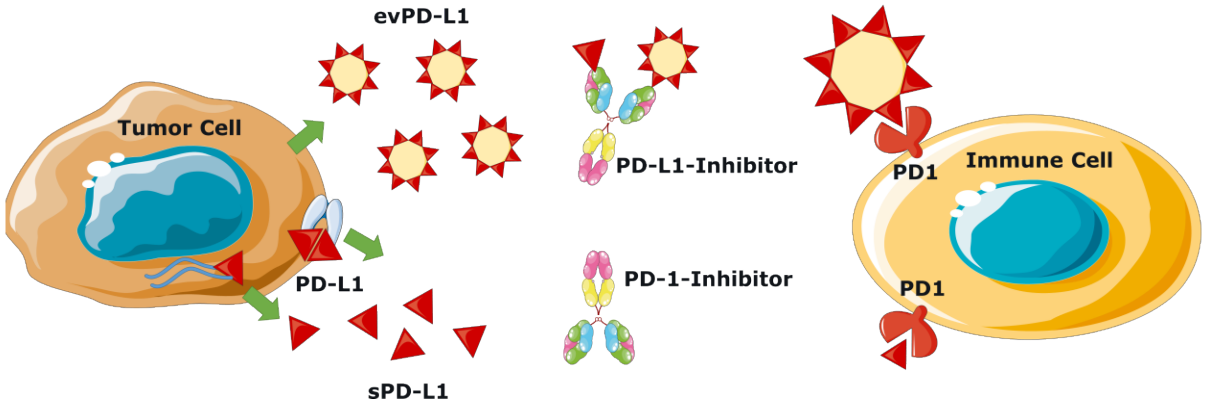

1.2 Therapeutic plasma exchange clears evPD-L1 and sPD-L1

Therapeutic plasma exchange (TPE) is a procedure in which blood is removed from the body and processed to eliminate non-diffusing substances confined to the plasma (Fig 2A). TPE is commonly used clinically to remove pathogenic antibodies in patients with severe autoimmune disease. In our pilot study of 24 patients, TPE significantly depleted sPD-L1 (Fig 2B) and evPD-L1 (Fig 4C) by 70.8% and 73.1%, respectively [16]. This depletion is compounded over three consecutive TPE sessions.

Fig 2 – Therapeutic Plasma Exchange (TPE) removes sPD-L1 and evPD-L1 from circulation.

(A) In TPE, blood is removed and centrifuged to remove substances, such as antibodies, from systemic circulation. (B,C) TPE removed an average of 70.8% of sPD-L1 and 73.1% of evPD-L1 from the blood in one session.

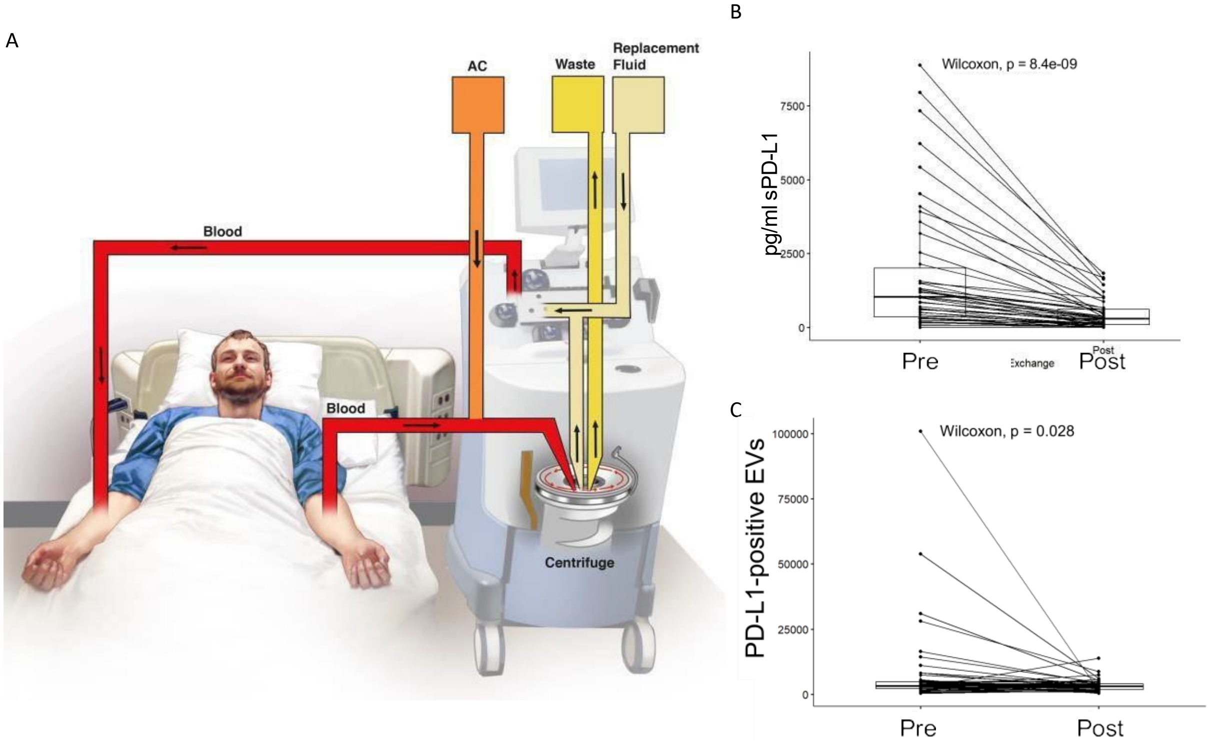

1.3 Defining the kinetics of sPD-L1 and evPD-L1 in melanoma

The *rationale* for this study is that its successful completion will define the kinetic quality of sPD-L1 and evPD-L1 depletion and regeneration in melanoma, without which a full Phase I clinical trial of TPE PD-(L)1 inhibitor rescue cannot be performed. Patients with advanced melanoma who progress on first-line ICI will be enrolled in this study (n=36). Patients with high sPD-L1 levels (> 1.7 ng/ml), ECOG 0-1, life expectancy > 6 months are eligible. After standard-of-care radiation therapy, eligible patients will undergo 3 days of TPE treatment, after which baseline sPD-L1 and evPD-L1 will be measured before standard-of-care immunotherapy is administered. Just prior to the next indicated immunotherapy 2-3 weeks later, sPD-L1 and evPD-L1 will be measured again and compared for regeneration.

2. Goals

2.1. Primary Goal

2.1.1. To determine the kinetics of sPD-L1 removal and regeneration by plasma exchange in patients with melanoma.

2.2. Secondary Goals

2.2.1. To observe response at 3 months after plasma exchange plus immunotherapy.

2.2.2. To observe ongoing response at approximate 3 month intervals after plasma exchange plus immunotherapy.

2.3. Correlative Research

2.3.1. To determine the effects of plasma exchange on immune cell function.

2.3.2. To observe the kinetics of extracellular vesicles (EVs) after plasma exchange in patients with melanoma.

3. Patient Eligibility

3.1. Inclusion Criteria

3.1.1. Age ≥18 years.

3.1.2. Histological confirmation of melanoma. Patients may have completed biopsy outside of Mayo Clinic, but there must be an internal review done to confirm diagnosis prior to confirming eligibility.

3.1.3. Measurable or non-measurable disease as defined in Section 11.0.Patient must meet one of these criteria.

3.1.4. ECOG Performance Status (PS) ≤ 3 (Appendix I).

3.1.5. sPD-L1 levels >1.7ng/ml by ELISA [17]

3.1.6. Negative pregnancy test done ≤7 days prior to radiation therapy, for women of childbearing potential only.

3.1.7. Provide written informed consent.

3.1.8. Willing to return to enrolling institution for follow-up (during the Active Monitoring Phase of the study).

3.1.9. Willing to provide blood samples for correlative research purposes (see Section 6).

3.2. Exclusion Criteria

3.2.1. Any of the following:

3.2.1.1. Persons taking a biotin supplement

3.2.1.2. sPD-L1 level <1.7ng/ml by ELISA [17]

3.2.1.3. Pregnant or nursing women

3.2.1.4. Men or women of childbearing potential who are unwilling to employ adequate contraception

4. Test Schedule

	Screening	Radiation Therapy	Therapeutic Plasma Exchange R, 4, 8	Observation/Follow up until 2 years	Progression
				post registration or progression (off study)	

Tests and procedures[8]	≤_30_ days prior to registration	Following Last Fraction of RT	Daily during plasma exchange (TPE)	Last plasma exchange (TPE)	- Pre-2nd IO[10]	At every clinically indicated follow up until progression[2]	
Toxicity Assessments - CTCAE v5.0 [S, 2]	X[6]	X[12]	X[5]		X[6]	X[6]	
Pregnancy test [1, S]	X						
Vascular Assessment	X[9]						
CBC for Apheresis machine set up [S]	X						
Research blood specimens (see Section 14.0) [R]	X	X[7, 3]		X[7, 3]	X		X[8]
Routine Imaging [S]						X	
Temporary central line placement (in patients without adequate venous access)[R]	X[11]						
Temporary central line removal (in patients with line) [R]				X			

Radiation Treatment = 1 to 10 fractions, up to 60 Gy total, clinician's choice
IO = Immunotherapy, clinician's choice pembrolizumab or nivolumab

1. For women of childbearing potential only. Must be done *≤30 days* prior to beginning Radiation Treatment as a standard of care procedure.
2. Follow up adverse event assessments/visits will be completed as per Radiation Oncology clinician's discretion, which generally takes place every 3-6 months until progression. Local oncologist's standard of care assessments are acceptable and will be assessed in Radiation Oncology.

3. Blood to be collected in Therapeutic Apheresis Treatment Unit – Charlton 8A.

4. The plasma exchange treatments to begin following completion of RT and will be done in 3 consecutive days.

5. Standard of care AE assessments during exchange will be completed daily by Study Apheresis RN, while doing plasma exchange treatment

6. To be assessed by Medical Oncology or Radiation Oncology using irAE toxicity assessment until progression. Additionally, baseline AE's can be performed after consent/registration as long as they are completed before treatment start.

7. Following the last fraction of RT (Pre-Initial TPE) and Post-Final TPE. Must have results available within 2 weeks of starting TPE for machine set up.

8. Any patient who progresses, during treatment or after completion, will go directly to Event Monitoring section 4.1. We will collect an additional blood draw if patient is willing.

9. Vein access for TPE must be determined by study apheresis technician, this will occur before TPE start.

10. 2 weeks post first IO if receiving **nivolumab**, 3 weeks post first IO if receiving **pembrolizumab**

11. Eligible, enrolled patients determined to require central line placement will undergo Mahurkar line placement by interventional radiology 48-72 hours prior to first TPE procedure. Central line will be removed at apheresis unit at completion of final plasma exchange.
12. To be collected at last management visit for Radiation Therapy

R Research funded (see Section 19.0)

S Standard of care

4.1 Event Monitoring Schedule

	**Observation/Follow-up[1] 2 years post registration**			
	6M	1Y	18M	2Y
Survival Status	X	X	X	X

1. If a patient is still alive 2 years after registration, no further follow-up is required.

5. Grouping Factors:

5.1. High versus low baseline evPD-L1 (cutoffs >1 million or 0.05%)

6. Registration Procedures

6.1. Registration

Patient will be registered (accrued) to the study when they have consented, met eligibility criteria and have been logged into the Research Participant Tracking System (Ptrax).

6.1.1. Verify the following procedures are completed prior to Registration to the study.

6.1.2. IRB approval at the registering institution

6.1.3. Existence of a signed informed consent

6.1.4. Patient eligibility complete

Note: Radiation Therapy on this protocol must be performed at Mayo Clinic Rochester under the supervision of a radiation oncologist.

7. Protocol Treatment

7.1. Radiation Therapy (RT) if indicated

Given per treating radiation oncologist discretion on days D1 through D5. The prescription dose is expected to be up to 60 Gy delivered in up to 5 fractions but not stipulated by the study. Treatment will be delivered daily except on weekends and holidays at the discretion of the treating radiation oncologist. Thus, D1 through D5 may not occur on consecutive days per protocol.

7.2. Line placement if indicated

In patients without adequate peripheral iv access as determined by apheresis unit nursing staff, a temporary Mahurkar central line will be placed by interventional radiology to enable therapeutic plasma exchange. This will be performed in patients requiring a line at least 48 hours prior to first therapeutic plasma exchange session. Line will be removed by trained apheresis unit staff after final therapeutic plasma exchange session.

7.3. Therapeutic plasma exchange (TPE)

Three sessions performed on consecutive days, with the first session starting on the last day of radiation therapy and the last session ending prior to immunotherapy. . This procedure is *not* performed prior to subsequent immunotherapy in future cycles.

7.4. Concurrent immunotherapy (IO)

According to clinician preference, either pembrolizumab or nivolumab will be given within the following five days after therapeutic plasma exchange (generally D7) . This immunotherapy will be continued per clinician judgement as clinically indicated and following standard of care guidelines until progression and/or adverse events requiring discontinuation.

Agent	Dose	Days administered	Pre-medication*
Pembrolizumab (Keytruda®)	200mg IV	Within 5 days of TPE	None
Nivolumab (Opdivo®)	240mg IV	Within 5 days of TPE	None

7.5. Follow up protocol

7.5.1. Follow-up imaging

Standard of care CT, PET, and/or MRI scans will be used as clinically indicated and ordered for follow up imaging.

7.6. Response assessment

7.6.1. Post-treatment tumor recurrence will be monitored with follow-up imaging to assess tumor response based on RECIST criteria until progression or death (or up to 2 years)

7.7. Outcomes

Patient-centric outcomes measured will include time to progression and time to death if applicable. These will be determined by chart review.

7.8. Acute and late toxicity monitoring

Toxicity will be measured by standard-of-care irAE forms. No known side-effect of plasma exchange constitutes an adverse event that is not anticipated by irAE monitoring.

7.9. Dose Modification Based on Adverse Event

Dose modification for immunotherapies in future cycles will be at the discretion of the treating physician.

7.10. Return to consenting institution if applicable

For this protocol, the patient must return to the consenting institution for evaluation at least every 182 days during treatment. Local treating physician evaluations, if done according to standard of care, are also acceptable after blood draws have been completed.

8. Dosage Modification Based on Adverse Events

8.1. Initial treatment with TPE carries low but possible risk of hypotension, infection, or excessive bleeding. Should any patient experience an adverse event related to plasma exchange, TPE will be discontinued.

8.2. Dose modification of pembrolizumab or nivolumab will be performed according to clinician preference over the course of the study. These changes are made at the discretion of the treating physician.

9. Ancillary Treatment/Supportive Care

9.1. Antiemetics

Antiemetics may be used at the discretion of the attending physician.

9.2. Premedication for chemotherapy will be given per institutional protocols.

9.3. No biotin-containing compounds will be administered or prescribed.

10. Adverse Event (AE) Monitoring and Reporting

10.1. Definitions

10.1.1. Adverse Event: Any untoward medical occurrence associated with the use of a drug in humans, whether or not considered drug related.

10.1.2. Serious Adverse Event: Adverse events are classified as serious or non-serious. Serious problems/events can be well defined and include:

10.1.2.1. Death

10.1.2.2. Life threatening adverse experience

10.1.2.3. Hospitalization

10.1.2.4. Inpatient, new, or prolonged; disability/incapacity

10.1.2.5. Persistent or significant birth defect/anomaly

10.1.2.6. and/or per protocol may be problems/events that in the option of the sponsor investigator may have adversely affected the rights, safety, or welfare of the subjects or others, or substantially comprised the research data. All adverse events that do not meet any of the criteria for serious, should be regarded as non-serious adverse events.

Unanticipated Problems Involving Risks to Subjects or Others (UPIRTSO)- Any unanticipated problem or adverse event that meets the following three criteria:

- Serious: Serious problems or events that results in significant harm, (which may be physical, psychological, financial, social, economic, or legal) or increased risk for the subject or others (including individuals who are not research subjects). These include: (1) death; (2) life threatening adverse experience; (3) hospitalization - inpatient, new, or prolonged; (4) disability/incapacity - persistent or significant; (5) birth defect/anomaly; (6) breach of confidentiality and (7) other problems, events, or new information (i.e. publications, DSMB reports, interim findings, product labeling change) that in the opinion of the local investigator may adversely affect the rights, safety, or welfare of the subjects or others, or substantially compromise the research data,

  AND

- Unanticipated: (i.e. unexpected) problems or events are those that are not already described as potential risks in the protocol, consent document, not listed in the Investigator's Brochure, or not part of an underlying disease. A problem or event is

"unanticipated" when it was unforeseeable at the time of its occurrence. A problem or event is

"unanticipated" when it occurs at an increased frequency or at an increased severity than expected, AND

- Related: A problem or event is "related" if it is possibly related to the research procedures.
- Preexisting Condition- A preexisting condition is one that is present at the start of the study. A preexisting condition should be recorded as an adverse event if the frequency, intensity, or the character of the condition worsens during the study period. At screening, any clinically significant abnormality should be recorded as a preexisting condition. At the end of the study, any new clinically significant findings/abnormalities that meet the definition of an adverse event must also be recorded and documented as an adverse event.

10.1.3. Suspected Adverse Reaction: Any adverse event for which there is a reasonable possibility that the drug or procedure caused the adverse event.

10.2. Recording Adverse Events/Treatment being studied

CTCAE term (AE description) and grade: The descriptions and grading scales found in the revised NCI Common Terminology Criteria for Adverse Events (CTCAE) version 5.0 will be utilized for AE reporting. All appropriate treatment areas should have access to a copy of the CTCAE version 5.0. A copy of the CTCAE version 5.0 can be downloaded from the CTEP web site: (http://ctep.cancer.gov/protocolDevelopment/electronic_applications/ctc.htm)

10.2.1. Adverse event monitoring and reporting is a routine part of every clinical trial. First, identify and grade the severity of the event using the CTCAE version 5.0. Next, determine whether the event is expected or unexpected and if the adverse event is related to the medical treatment or procedure. With this information, determine whether the event must be reported as an expedited report (see Section 10.25).

Expedited and routine reports are to be completed within the timeframes and via the mechanisms specified in Sections 10.4 and 10.6. All expected AE reports must also be sent to the local Institutional Review Board (IRB) according to local IRB's policies and procedures.

10.2.1.1.1.1. When assessing whether an adverse event (AE) is related to a medical agent(s) medical or procedure, the following attribution categories are utilized:

Definite - The AE is clearly related to the agent(s)/procedure.

Probable - The AE is likely related to the agent(s)/procedure.

Possible - The AE may be related to the agent(s)/procedure.

Unlikely - The AE is doubtfully related to the agent(s)/procedure.

Unrelated - The AE is clearly NOT related to the agent(s)/procedure.

10.2.2. Exceptions to Expedited Reporting For Immunotherapy Related Toxicities

An expedited report may not be required for specific Grade 1, 2 and 3 serious Adverse Events. Any protocol specific reporting procedures MUST BE SPECIFIED BELOW and will supersede the standard Expedited Adverse Event Reporting Requirements**:** **Hospitalizations for reasons deemed to be disease related will not be reported.**

CTCAE System Organ Class (SOC)	Adverse event/ Symptoms	CTCAE Grade at which the event will not be reported in an expedited manner[1]
General disorders and administrations site conditions	Fatigue	*≤Grade 3*
	Malaise	*≤Grade 3*
Skin and subcutaneous/ Tissue Disorders	Alopecia	*≤Grade 4*

[1] These exceptions only apply if the adverse event does not result in hospitalization. If the adverse event results in hospitalization, then the standard expedited adverse events reporting requirements must be followed.
Specific protocol exceptions to expedited reporting should be reported expeditiously by investigators **ONLY** if they exceed the expected grade of the event.

[1] These exceptions only apply if the adverse event does not result in hospitalization. If the adverse event results in hospitalization, then the standard expedited adverse events reporting requirements must be followed.
Specific protocol exceptions to expedited reporting should be reported expeditiously by investigators **ONLY** if they exceed the expected grade of the event.

10.3. Other Required Reporting

10.3.1. Unanticipated Problems Involving Risks to Subjects or Others (UPIRTSOS)

Unanticipated Problems Involving Risks to Subjects or Others (UPIRTSOS) in general, include any incident, experience, or outcome that meets **all** of the following criteria:

1. Unexpected (in terms of nature, severity, or frequency) given (a) the research procedures that are described in the protocol-related documents, such as the IRB-approved research protocol and informed consent document; and (b) the characteristics of the subject population being studied;
2. Related or possibly related to participation in the research (in this guidance document, possibly related means there is a reasonable possibility that the incident, experience, or outcome may have been caused by the procedures involved in the research); and
3. Suggests that the research places subjects or others at a greater risk of harm (including physical, psychological, economic, or social harm) than was previously known or recognized.

Some unanticipated problems involve social or economic harm instead of the physical or psychological harm associated with adverse events. In other cases, unanticipated problems place subjects or others at increased *risk* of harm, but no harm occurs.

Note: If there is no language in the protocol indicating that pregnancy is not considered an adverse experience for this trial, and if the consent form does not indicate that subjects should not get pregnant/impregnate others, then any pregnancy in a subject/patient or a male patient's partner (spontaneously reported) which occurs during the study or within

10.3.2. Death

Note: A death on study requires both routine and expedited reporting regardless of causality, unless as noted below. Attribution to treatment or other cause must be provided.

Any death occurring within 30 days of the last dose of TPE, regardless of attribution to an agent/intervention under an IND/IDE requires expedited reporting within 24-hours.

Any death occurring greater than 30 days with an attribution of possible, probable, or definite to an agent/intervention under an IND/IDE requires expedited reporting within 24-hours.

Reportable categories of Death

- Death attributable to a CTCAE term.
- Death Neonatal: A disorder characterized by cessation of life during the first 28 days of life.
- Death NOS: A cessation of life that cannot be attributed to a CTCAE term associated with Grade 5.
- Sudden death NOS: A sudden (defined as instant or within one hour of the onset of symptoms) or an unobserved cessation of life that cannot be attributed to a CTCAE term associated with Grade 5.
- Death due to progressive disease should be reported as **Grade 5 "Neoplasms benign, malignant and unspecified (including cysts and polyps) – Other (Progressive Disease)"** under the system organ class (SOC) of the same name. Evidence that the death was a manifestation of underlying disease (e.g., radiological changes suggesting tumor growth or progression: clinical deterioration associated with a disease process) should be submitted.

10.4. Required Routine Reporting

10.4.1. Toxicity Evaluations

Pretreatment symptoms/conditions to be graded at baseline and adverse events to be graded at each evaluation.

Grading is per CTCAE v5.0 **unless** alternate grading is indicated in the table below: (this table outlines the AE's you want to have assessed at each study visit the patient has)

CTCAE System/Organ/Class (SOC)	Adverse event/Symptoms	Baseline	Each evaluation
Gastrointestinal	*Diarrhea*	x	x
Dermatologic	*Rashes*	x	x

CTCAE System/Organ/Class (SOC)	Adverse event/Symptoms	Baseline	Each evaluation
Respiratory	*Pneumonitis*	x	x
Adrenal	*Hypophysitis*	x	x
Rheumatologic	*Myalgias, arthralgias*	x	x
Liver	*Hepatic failure*	x	x

10.5. Monitoring and Auditing

The investigator will permit study-related monitoring, audits, and inspections by the IRB, the sponsor, and government regulatory agencies, of all study related documents (e.g. source documents, regulatory documents, data collection instruments, study data etc.). The investigator will ensure the capability for inspections of applicable study-related facilities (e.g. pharmacy, diagnostic laboratory, etc.). Participation as an investigator in this study implies acceptance of potential inspection by government regulatory authorities and applicable compliance offices

10.5.1. Medical Monitoring

It is the responsibility of the Principal Investigator to oversee the safety of the study at his/her site. This safety monitoring will include careful assessment and appropriate reporting of adverse events as noted above, as well as the construction and implementation of a site data and safety-monitoring plan (see section 10.5 “Monitoring and Auditing”). Medical monitoring will include a regular assessment of the number and type of serious adverse events. “Any serious adverse events will be followed up by the sentinel event reporting procedure”

10.5.2. Internal Data and Safety Monitoring Board

The trial will be reviewed by the Cancer Center Auditing area on a bi-annual or yearly basis dependent on random study selection to assess accrual, adverse events, and any endpoint problems. Any safety issues requiring protocol changes will be communicated through protocol amendments.

11. Treatment Evaluation/Measurement of Effect

Response and progression will be evaluated in this study using the new international criteria proposed by the revised Response Evaluation Criteria in Solid Tumors (RECIST) guidelines (version 1.1) [18]. Changes in the largest diameter (unidimensional measurement) of the tumor lesions and the short axis measurements in the case of lymph nodes are used in the RECIST guideline.

11.1. Schedule of Evaluations:

11.1.1. For the purposes of this study, patients should be reevaluated every 3-6 months according to clinical judgment.

11.2. Definitions of Measurable and Non-Measurable Disease

11.2.1. Measurable Disease

11.2.1.1. A non-nodal lesion is considered measurable if its longest diameter can be accurately measured as ≥2.0 cm with chest x- ray, or as ≥1.0 cm with CT scan, CT component of a PET/CT, or MRI.

11.2.1.2. A superficial non-nodal lesion is measurable if its longest diameter is ≥ 1.0 cm in diameter as assessed using calipers (e.g. skin nodules) or imaging. In the case of skin lesions, documentation by color photography, including a ruler to estimate the size of the lesion, is recommended.

11.2.1.3. A malignant lymph node is considered measurable if its short axis is >1.5 cm when assessed by CT scan (CT scan slice thickness recommended to be no greater than 5 mm).

NOTE: Tumor lesions in a previously irradiated area are not considered measurable disease.

11.2.2. Non-Measurable Disease

11.2.2.1. All other lesions (or sites of disease) are considered non- measurable disease, including pathological nodes (those with a short axis ≥1.0 to <1.5 cm). Bone lesions, leptomeningeal disease, ascites, pleural/pericardial effusions, lymphangitis cutis/pulmonis, inflammatory breast disease, and abdominal masses (not followed by CT or MRI), are considered as non- measurable as well.

Note: 'Cystic lesions' thought to represent cystic metastases can be considered as measurable lesions, if they meet the definition of measurability described above. However, if non-cystic lesions are present in the same patient, these are preferred for selection as target lesions. In addition, lymph nodes that have a short axis <1.0 cm are considered non-pathological (i.e., normal) and should not be recorded or followed.

11.3. Guidelines for Evaluation of Measurable Disease

11.3.1. Measurement Methods:

- All measurements should be recorded in metric notation (i.e., decimal fractions of centimeters) using a ruler or calipers.
- The same method of assessment and the same technique must be used to characterize each identified and reported lesion at baseline and during follow-up. For patients having only lesions measuring at least 1 cm to less than 2 cm must use CT imaging for both pre- and post- treatment tumor assessments.
- Imaging-based evaluation is preferred to evaluation by clinical examination when both methods have been used at the same evaluation to assess the antitumor effect of a treatment.

11.3.2. Acceptable Modalities for Measurable Disease:

- Conventional CT and MRI: This guideline has defined measurability of lesions on CT scan based on the assumption that CT slice thickness is 5 mm or less. If CT scans have slice thickness greater than 5 mm, the minimum size for a measurable lesion should be twice the slice thickness.

- As with CT, if an MRI is performed, the technical specifications of the scanning sequences used should be optimized for the evaluation of the type and site of disease. The lesions should be measured on the same pulse sequence. Ideally, the same type of scanner should be used and the image acquisition protocol should be followed as closely as possible to prior scans. Body scans should be performed with breath-hold scanning techniques, if possible.

- PET-CT: If the site can document that the CT performed as part of a PET-CT is of identical diagnostic quality to a diagnostic CT (with IV and oral contrast), then the CT portion of the PET-CT can be used for RECIST measurements and can be used interchangeably with conventional CT in accurately measuring cancer lesions over time.

- Chest X-ray: Lesions on chest x-ray are acceptable as measurable lesions when they are clearly defined and surrounded by aerated lung. However, CT scans are preferable.

- Physical Examination: For superficial non-nodal lesions, physical examination is acceptable, but imaging is preferable, if both can be done. In the case of skin lesions, documentation by color photography, including a ruler to estimate the size of the lesion, is recommended.

- FDG-PET: FDG-PET scanning is allowed to complement CT scanning in assessment of progressive disease [PD] and particularly possible 'new' disease. A 'positive' FDG-PET scanned lesion is defined as one which is FDG avid with an update greater than twice that of the surrounding tissue on the attenuation corrected image; otherwise, an FDG-PET scanned lesion is considered 'negative.' New lesions on the basis of FDG-PET imaging can be identified according to the following algorithm:

    a. Negative FDG-PET at baseline with a positive FDG-PET at follow- up is a sign of PD based on a new lesion.

    b. No FDG-PET at baseline and a positive FDG-PET at follow-up:

        i. If the positive FDG-PET at follow-up corresponds to a new site of disease confirmed by CT, this is PD.

        ii. If the positive FDG-PET at follow-up is not confirmed as a new site of disease on CT at the same evaluation, additional follow- up CT scans (i.e., additional follow-up scans at least 4 weeks later) are needed to determine if there is truly progression occurring at that site. In this situation, the date of PD will be the date of the initial abnormal PDG-PET scan.

        iii If the positive FDG-PET at follow-up corresponds to a pre-existing site of disease on CT that is not progressing on the basis of the anatomic images, it is not classified as PD.

11.3.3. Measurement at Follow-up Evaluation:

- In the case of stable disease (SD), follow-up measurements must have met the SD criteria at least once after study entry at a minimum interval of 6 weeks.

- The cytological confirmation of the neoplastic origin of any effusion that appears or worsens during treatment when the

measurable tumor has

met criteria for response or stable disease is mandatory to differentiate between response or stable disease (an effusion may be a side effect of the treatment) and progressive disease.

- Cytologic and histologic techniques can be used to differentiate between PR and CR in rare cases (e.g., residual lesions in tumor types such as germ cell tumors, where known residual benign tumors can remain.)

11.4. Measurement of Effect

11.4.1. Target Lesions and Target Lymph Nodes

- Measurable lesions (as defined in Section 11.2.1) up to a maximum of 5 lesions, representative of all involved organs, should be identified as "Target Lesions" and recorded and measured at baseline. These lesions can be non-nodal or nodal (as defined in 11.2.1), where no more than 2 lesions are from the same organ and no more than 2 malignant nodal lesions are selected.

  Note: If fewer than 5 target lesions and target lymph nodes are identified (as there often will be), there is no reason to perform additional studies beyond those specified in the protocol to discover new lesions.

- Target lesions and target lymph nodes should be selected on the basis of their size, be representative of all involved sites of disease, but in addition should be those that lend themselves to reproducible repeated measurements. It may be the case that, on occasion, the largest lesion (or malignant lymph node) does not lend itself to reproducible measurements in which circumstance the next largest lesion (or malignant lymph node) which can be measured reproducibly should be selected.

- Baseline Sum of Dimensions (BSD): A sum of the longest diameter for all target lesions plus the sum of the short axis of all the target lymph nodes will be calculated and reported as the baseline sum of dimensions (BSD). The BSD will be used as reference to further characterize any objective tumor response in the measurable dimension of the disease.

- Post-Baseline Sum of the Dimensions (PBSD): A sum of the longest diameter for all target lesions plus the sum of the short axis of all the target lymph nodes will be calculated and reported as the post-baseline sum of dimensions (PBSD). If the radiologist is able to provide an actual measure for the target lesion (or target lymph node), that should

be recorded, even if it is below 0.5 cm. If the target lesion (or target lymph node) is believed to be present and is faintly seen but too small to measure, a default value of 0.5 cm should be assigned. If it is the opinion of the radiologist that the target lesion or target lymph node has likely disappeared, the measurement should be recorded as 0 cm.

- The minimum sum of the dimensions (MSD) is the minimum of the BSD and the PBSD.

11.4.2. Non-Target Lesions & Non-Target Lymph Nodes

Non-measurable sites of disease (Section 11.2.2) are classified as non-target lesions or non-target lymph nodes and should also be recorded at baseline. These lesions and lymph nodes should be followed in accord with the above.

11.4.3. Response Criteria

11.4.3.1. All target lesions and target lymph nodes followed by CT/MRI/ PET-CT/Chest X-ray/physical examination must be measured on re-evaluation at evaluation times specified in Section 11.1. Specifically, a change in objective status to either a PR or CR cannot be done without re-measuring target lesions and target lymph nodes.

Note: Non-target lesions and non-target lymph nodes should be evaluated at each assessment, especially in the case of first response or confirmation of response. In selected circumstances, certain non-target organs may be evaluated less frequently. For example, bone scans may need to be repeated only when complete response is identified in target disease or when progression in bone is suspected.

11.4.3.2. Evaluation of Target Lesions

Complete Response (CR):
All of the following must be true:

a. Disappearance of all target lesions.

b. Each target lymph node must have reduction in short axis to <1.0 cm.

Partial Response (PR):
At least a 30% decrease in PBSD (sum of the longest diameter for all target lesions plus the sum of the

short axis of all the target lymph nodes at current evaluation) taking as reference the BSD (*see* Section 11.4.1).

Progression (PD):
At least one of the following must be true:

a. At least one new malignant lesion, which also includes any lymph node that was normal at baseline (< 1.0 cm short axis) and increased to ≥ 1.0 cm short axis during follow-up.

b. At least a 20% increase in PBSD (sum of the longest diameter for all target lesions plus the sum of the short axis of all the target lymph nodes at current evaluation) taking as reference the MSD (Section 11.4.1). In addition, the PBSD must also demonstrate an absolute increase of at least 0.5 cm from the MSD.

c. See Section 11.32 for details in regards to the requirements for PD via FDG-PET imaging.

Stable Disease (SD):
Neither sufficient shrinkage to qualify for PR, nor sufficient increase to qualify for PD taking as reference the MSD.

11.4.3.3. Evaluation of Non-Target Lesions & Non-Target Lymph

Nodes Complete Response (CR):
All of the following must be true:

a. Disappearance of all non-target lesions.

b. Each non-target lymph node must have a reduction in short axis to <1.0 cm.

Non-CR/Non-PD: Persistence of one or more non-target lesions or non-target lymph nodes.

Progression (PD): At least one of the following must be true:

a. At least one new malignant lesion, which also includes any lymph node that was normal at baseline (< 1.0 cm short axis) and increased to ≥ 1.0 cm short axis during follow-up.

b. Unequivocal progression of existing non-target lesions and non-target lymph nodes. (NOTE: Unequivocal progression should not normally trump target lesion and target lymph node status. It must be representative of overall disease status change.)

c. See Section 11.3.2 for details in regards to the requirements for PD via FDG-PET imaging.

11.4.4. Overall Objective Status

The overall objective status for an evaluation is determined by combining the patient's status on target lesions, target lymph nodes, non-target lesions, non-target lymph nodes, and new disease as defined in the following table:

For Patients with Measurable Disease

Target Lesions & Target Lymph Nodes	Non-Target Lesions & Non-Target Lymph Nodes	New Sites of Disease	Overall Objective Status
CR	CR	No	CR
CR	Non-CR/Non-PD	No	PR
PR	CR<br>Non-CR/Non-PD	No	PR
CR/PR	Not All Evaluated*	No	PR**
SD	CR<br>Non-CR/Non-PD<br>Not All Evaluated*	No	SD
Not all Evaluated	CR<br>Non-CR/Non-PD<br>Not All Evaluated*	No	Not Evaluated (NE)
PD	Unequivocal PD<br>CR<br>Non-CR/Non-PD<br>Not All Evaluated*	Yes or No	PD

Target Lesions & Target Lymph Nodes	Non-Target Lesions & Non-Target Lymph Nodes	New Sites of Disease	Overall Objective Status
CR/PR/SD/PD/Not all Evaluated	Unequivocal PD	Yes or No	PD
CR/PR/SD/PD/Not all Evaluated	CR<br>Non-CR/Non-PD<br>Not All Evaluated*	Yes	PD

*See Section 11.4.3.1
** NOTE: This study uses the protocol RECIST v1.1 template dated 2/16/2011. For data collection and analysis purposes the objective status changed from SD to PR in the NCCTG protocol RECIST v1.1 template as of 2/16/2011 and to match RECIST v1.1 requirements.

11.4.5. Symptomatic Deterioration

Patients with global deterioration of health status requiring discontinuation of treatment without objective evidence of disease progression at that time, and not either related to study treatment or other medical conditions, should be reported as PD due to “symptomatic deterioration.” Every effort should be made to document the objective progression even after discontinuation of treatment due to symptomatic deterioration. A patient is classified as having PD due to “symptomatic deterioration” if any of the following occur that are not either related to study treatment or other medical conditions:

- Weight loss >10% of body weight.
- Worsening of tumor-related symptoms.
- Decline in performance status of >1 level on ECOG scale.

12. Descriptive Factors

12.1. Prior treatment: 1 vs 2 vs 3+

12.2. Disease stage

12.3. Patient age

12.4. Other medical problems

12.5. Baseline sPD-L1

12.6. Baseline evPD-L1

13. Treatment/Follow–up Decision at Evaluation of Patient

13.1. A patient is deemed *ineligible* if after registration, it is determined that at the time of registration, the patient did not satisfy each and every eligibility criteria for study entry. The patient will go off study.

13.1.1. If the patient received CT, MR, and/or PET for radiotherapy planning, all data up until the point of confirmation of ineligibility must be submitted.

13.1.2. If the patient never received plasma exchange, on-study material must be submitted. No additional follow up is necessary.

13.2. Those patients who will not receive any radiation treatment or who will receive radiation treatment elsewhere will go off study.

13.3. Patients who are CR, PR, REGR, or SD will continue to obtain imaging at each clinically indicated follow up for up to *2 years from registration*.

13.4. Patients who develop PD or withdraw from further follow up assessments will be followed for survival only.

13.5. Inevaluable patients: If a patient fails to complete therapy for reasons other than toxicity, the patient will be regarded as inevaluable and will be replaced.

14. Body Fluid Biospecimens

Correlative Study (Section 4.0 for more information)	Mandatory or Optional	Blood or Body Fluid being Collected	Type of Collection Tube (color of tube top)	Volume to collect per tube (# of tubes to be collected)	Visit 1 *(Screening)*	Visit 2 (*Pre- Initial TPE)*	Visit 3 *(Post-Final TPE*	Visit 4 *(Pre-2nd IO)*	Visit 5 At time of progression	Process at site? (Yes or No)	Temp/Cond/ Storage/ Shipping
Pharmacokinetics, Circulating EVs, PBMCs	Mandatory	Serum	Yellow	6mL (6)	X	X	X	X	X	No	Ambient

Waste	Mandatory	Plasma exchange waste product	Waste bag	Up to 800cc (1)		X				No	Ambient

14.1. Collection and Processing

Specimens will be collected at the following time points:

- #1 Pre-SBRT (or one week prior to immunotherapy)
- #2 Pre-Initial TPE (for selected patients with sPD-L1 levels above cutoff)
- #3 Post-Final TPE
- #4 2-3 week follow-up at standard clinical appointment prior to next round of immunotherapy
- #5 At time of progression

14.1.1. 6-6ML tubes, 36 ml will be collected in citrate yellow-toped tubes at each timepoint. The tubes should be gently inverted 7 to 10 times to thoroughly mix the samples. These samples will be processed within 7 days of collection for sPD-L1, extracellular vesicles, and other metabolites using ELISA and flow cytometry per lab protocols. All other samples must be labeled for processing within one week. Citrate tubes will be provided for this project.

15. 0 Drug Information

15.1. **Pembrolizumab (Keytruda®)** – Standard of care treatment allowed in this study.

15.1.1. Approved for treatment of advanced melanoma (unresectable or metastatic)

15.1.2. Dose 200mg IV every 3 weeks until disease progression or unacceptable toxicity.

15.1.3. Class: Immune checkpoint inhibitor (ICI), monoclonal programmed death receptor-1 (PD-1) blocking human monoclonal antibody.

15.1.4. FDA approval for recurrent/metastatic cervical cancer, advanced endometrial carcinoma, recurrent locally advanced/metastatic esophageal or gastric cancer, unresectable/recurrent/metastatic head and neck squamous cell cancer, advanced hepatocellular carcinoma, relapsed/refractory classical Hodgkin lymphoma, recurrent/metastatic Merkel cell carcinoma, stage III or IV non-small cell lung cancer, relapsed/refractory mediastinal large B-cell lymphoma, advanced renal cell carcinoma, metastatic small cell lung cancer, high-risk locally advanced/metastatic urothelial carcinoma, and unresectable/metastatic microsatellite instability-high cancers of any kind [19].

15.1.5. Adverse events related to immune-related treatments (irAE) will be collected at each follow-up visit. Serious irAE include pneumonitis, colitis, hepatitis, nephritis/renal dysfunction, hypothyroidism/hyperthyroidism.

15.1.6. Pharmacokinetics: administered intravenously, clearance difference not considered to be clinically important.

15.2. **Nivolumab (Opdivo®)**– Standard of care treatment allowed in this study.

15.2.1. Approved for treatment of advanced melanoma (unresectable or metastatic)

15.2.2. Dose 240mg IV every 2 weeks until disease progression or unacceptable toxicity.

15.2.3. Class: Immune checkpoint inhibitor (ICI), monoclonal programmed death receptor-1 (PD-1) blocking human monoclonal antibody.

15.2.4. FDA approval for MSI-high colorectal cancer, recurrent/metastatic head and neck SCC, hepatocellular carcinoma, classical Hodgkin lymphoma,

unresectable/metastatic melanoma, advanced renal cell carcinoma, metastatic small cell lung cancer, and locally advanced/metastatic urothelial carcinoma [20].

15.2.5. Adverse events related to immune-related treatments (irAE) will be collected at each follow-up visit. Serious irAE include pneumonitis, colitis, hepatitis, nephritis/renal dysfunction, hypothyroidism/hyperthyroidism.

15.2.6. Pharmacokinetics: administered intravenously, clearance unchanged by dose. 30- minute infusion comparable to 60-minute infusion. Clearance increased by 20% in the presence of anti-nivolumab antibodies.

16.0 Statistical Considerations and Methodology

16.1 Overview and Study Design:

This is a feasibility trial that seeks to assess feasibility and kinetics of the plasma exchange approach in these patients. In addition, we plan to assess response rate, overall survival, progression-free survival, adverse events, and correlative analysis.

16.1.1 Primary Endpoint

The primary endpoint of this trial is to assess the feasibility and kinetics for this treatment approach. Feasibility will be assessed by being able to complete the study accrual in a reasonable time period, where if we cannot enroll all 20 patients within 2 years, we'll assume this approach isn't feasible. Kinetics of sPD-L1 production in melanoma will be assessed in an exploratory fashion by assessing the change in the sPD-L1 levels between different time points of interest. Since this feasibility study is small and exploratory, no power calculations are needed. Additional comparator substances or potentially pathogenic tumor substances will also be measured for comparison.

16.1.2 Sample Size

Eighteen eligible patients will be accrued onto this feasibility study. We anticipate accruing an additional 2 patients to account for ineligibility, cancellation, major treatment violation, or other reasons. Therefore, maximum accrual is expected to be 20 registered patients. To accrue 20 patients that exhibit sPD-L1 above our cutoff (usually ~35% of patients), we expect to pre-register around 60 patients total.

16.1.3 Accrual Time and Study Duration

The anticipated registration rate is approximately 2 patients per month. Therefore, the accrual period for this study is expected to be approximately 10 months. The final analysis can begin approximately 24 months after the trial begins, i.e. as soon as the last patient has been followed for at least 12 months plus time for data entry and clean-up.

16.2 Data and Safety Monitoring

The principal investigator(s) and the study statistician will review the study at least monthly to identify accrual, adverse event, and any endpoint problems that might be developing. The trial is monitored continually by the study team who are notified of every grade 4 and 5 event in real time. The Mayo Clinic Cancer Center (MCCC) Data Safety Monitoring Board (DSMB) is responsible for reviewing accrual and safety data for this trial at least twice a year, based on reports provided by the MCCC Statistical Office. Any safety

issues requiring protocol changes are communicated through protocol amendments.

Adverse Event Stopping Rule: Based on previous experience with this disease, we expect approximately 10% of patients to experience Grade 4+ adverse events. If at any time, 3 of the first 10 registered patients (or 30% or more beyond 10 registered patients) have experienced any Grade 4 or 5 adverse event (at least possibly related to the study treatment), accrual to the study will be suspended to allow for a full review of the data. Each grade 5 event will be reviewed on a case by case basis in a real time fashion to determine whether study accrual should be suspended. After consideration by the study team [ie, Study Chair(s), Statistician, Operations Office, etc] and consultation with representatives at the primary Internal Review Board (IRB) affiliated with the Operations Office, a decision will be made as to whether and how the study will proceed.

16.3. Analysis Plan

16.3.1. Primary Endpoint

For feasibility, we'll just keep track of the accrual and accrual rate. We're hoping for fast accrual, where we can register up to 2 patients per month. Feasibility will be assessed by being able to complete the study accrual in a reasonable time period, where if we can't register all 20 patients within 2 years, we'll assume this approach isn't feasible.

We'll also determine the kinetics of sPD-L1 removal and regeneration by plasma exchange in patients with melanoma. Graphical methods and descriptive statistics will be used to explore this endpoint. The Wilcoxon Signed-Rank test will be used to assess the change in the sPD-L1 levels over time across the different timepoints of interest (pre vs. post initial plasma exchange therapy; post plasma vs. 2 to 3-week follow-up; pre vs. 2 to 3-week follow-up timepoint). The hypothesis is that the sPD-L1 levels will decrease with the use of plasma exchange treatment. The hope is that this decrease will be maintained throughout the first 2 to 3 weeks of follow-up and beyond. Other potentially pathogenic tumor factors will also be measured and compared for correlated studies.

16.3.2. Definition and Analyses of Secondary Endpoints

16.3.2.1. Overall Response rate is the proportion of patients with a tumor response (PR or CR at least 4 weeks apart). All patients meeting the eligibility criteria who have signed a consent form and have begun treatment will be evaluable for the response rate.

16.3.2.2. Progression-free survival (PFS) is defined as the time from registration to the first of either disease progression or death from any cause. Patients who receive the study drug, but then never return for an evaluation will be censored on their last follow-up date. PFS will be estimated using the method of Kaplan-Meier.

16.3.2.3. Overall survival (OS) is defined as the time from registration to death from any cause. OS will be estimated using the method of Kaplan-Meier.

16.3.3. Adverse Events

All patients that have initiated treatment will be considered evaluable for adverse event (AE) analyses. The maximum grade for each type of AE will be recorded for each patient, and frequency tables will be reviewed to determine AE patterns.

16.3.4. Analysis of Translational Component

For this correlative analysis, we will determine the effects of plasma exchange on immune cell function, observe the kinetics of extracellular vesicles (EVs) after plasma exchange in patients with melanoma, and associate the kinetics with clinical outcome data (RR, OS, PFS). Associations of categorical data will be assessed using Fisher exact tests. Associations of continuous data with binary data will be assessed using standard Wilcoxon Rank-Sum tests. Assessment of the change in continuous data over time will be done using the Wilcoxon Signed-Rank test. Kaplan-Meier methods and the log-rank test will be used for time-to-event data. This translational study is considered exploratory and hypothesis generating due to the small proposed sample size for this study.

16.4. Inclusion of Women and Minorities

16.4.1. Study availability

This study will be available to all eligible patients, regardless of gender, race or ethnic origin.

16.4.2. Differential effects by race or ethnicity

There is no information currently available regarding differential effects of this regimen in subsets defined by gender, race or ethnicity, and there is no reason to expect such differences to exist. Therefore, although the planned analysis will, as always, look for differences in treatment effect based on racial groupings, the sample size is not increased in order to provide additional power for subset analyses.

16.4.3. Study population

Based on prior studies involving similar disease sites, we expect about 20% of patients will be classified as minorities by race and 5% will be women. Expected sizes (per study design) of racial by gender subsets are shown in the following table:

Accrual Estimates by Gender/Ethnicity/Race

Accrual Targets			
	Sex/Gender		
**Ethnic Category**	**Females**	**Males**	**Total**
Hispanic or Latino	2	2	4
Not Hispanic or Latino	8	8	16
**Ethnic Category: Total of all subjects**	**10**	**10**	**20**
**Racial Category**			
American Indian or Alaskan Native	0	0	0
Asian	1	1	2
Black or African American	1	1	2
Native Hawaiian or other Pacific Islander	0	0	0
White	8	8	16
**Racial Category: Total of all subjects**	**10**	**10**	**20**

Ethnic Categories: **Hispanic or Latino** – a person of Cuban, Mexican, Puerto Rican, South or Central American, or other Spanish culture or origin, regardless of race. The term "Spanish origin" can also be used in addition to "Hispanic or Latino."

Not Hispanic or Latino

Racial Categories: **American Indian or Alaskan Native** – a person having origins in any of the original peoples of North, Central, or South America, and who maintains tribal affiliations or community attachment.

Asian – a person having origins in any of the original peoples of the Far East, Southeast Asia, or the Indian subcontinent including, for example, Cambodia, China, India, Japan, Korea, Malaysia, Pakistan, the Philippine Islands, Thailand, and Vietnam. (Note: Individuals from the Philippine Islands have been recorded as Pacific Islanders in previous data collection strategies.)

Black or African American – a person having origins in any of the black racial groups of Africa. Terms such as "Haitian" or "Negro" can be used in addition to "Black or African American."

Native Hawaiian or other Pacific Islander – a person having origins in any of the original peoples of Hawaii, Guam, Samoa, or other Pacific Islands.

White – a person having origins in any of the original peoples of Europe, the Middle East, or North Africa.

17. Pathology Considerations/Tissue

Biospecimens No tissue Biospecimens.

18. Records and Data Collection Procedures

18.1 Data Handling and Record Keeping

18.1.1 Confidentiality

Information about study subjects will be kept confidential and managed according to the requirements of the Health Insurance Portability and Accountability Act of 1996 (HIPAA). Those regulations require a signed subject authorization informing the subject of the following:

- What protected health information (PHI) will be collected from subjects in this study
- Who will have access to that information and why
- Who will use or disclose that information
- The rights of a research subject to revoke their authorization for use of their PHI.

In the event that a subject revokes authorization to collect or use PHI, the investigator, by regulation, retains the ability to use all information collected prior to the revocation of subject authorization. For subjects that have revoked authorization to collect or use PHI, attempts should be made to obtain permission to collect at least vital status (long term survival status that the subject is alive) at the end of their scheduled study period.

18.1.2 Source Documents

Source data is all information, original records of clinical findings, observations, or other activities in a clinical trial necessary for the reconstruction and evaluation of the trial. Source data are contained in source documents. Examples of these original documents, and data records include: hospital records, clinical and office charts, laboratory notes, memoranda, subjects' diaries or evaluation checklists, pharmacy dispensing records, recorded data from automated instruments, copies or transcriptions certified after verification as being accurate and complete, microfiches, photographic negatives, microfilm or magnetic media, x-rays, subject files, and records kept at the pharmacy, at the laboratories, and at medico-technical departments involved in the clinical trial. Source documents are kept in a secure location that is locked and requires approved access.

18.1.3 Case Report Forms

The study case report form (CRF) is the primary data collection instrument for the study. All data requested on the CRF must be recorded. All missing data must be explained. All data will be entered into electronic case report forms (eCRF's)

through the Medidata Rave system. Case report forms will be automatically rolled out based on a predetermined and visit-based schedule to improve study staff workflow and data quality. Data will be exported nightly to a secure FTP for analysis and reporting.

18.1.4 Records Retention

The investigator will maintain records and essential documents related to the conduct of the study. These will include subject case histories and regulatory documents.

The investigator will retain the specified records and reports as outlined in the Mayo Clinic Research Policy Manual–"Retention of and Access to Research Data Policy" http://mayocontent.mayo.edu/research-policy/MSS_669717

19. Budget

19.1. Costs charged to patient: routine clinical care including SBRT, pembrolizumab or nivolumab depending on physician preference.

19.2. Tests to be research funded: Therapeutic Plasma Exchange (TPE), sPD-L1 ELISA, EV nanoflow, any other tests of potentially pathogenic or comparator substances in blood.

20. References

[1] M. Aitken, M. Kleinrock, A. Simorellis, and D. Nass, "Global Oncology Trends 2018," London, 2018.

[2] A. Haslam and V. Prasad, "Estimation of the Percentage of US Patients With Cancer Who Are Eligible for and Respond to Checkpoint Inhibitor Immunotherapy Drugs," *JAMA Netw. open*, vol. 2, no. 5, p. e192535, May 2019.

[3] J. S. O'Donnell, G. V Long, R. A. Scolyer, M. W. L. Teng, and M. J. Smyth, "Resistance to PD1/PDL1 checkpoint inhibition.," *Cancer Treat. Rev.*, vol. 52, pp. 71–81, Jan. 2017.

[4] J. Zhou *et al.*, "Soluble PD-L1 as a Biomarker in Malignant Melanoma Treated with Checkpoint Blockade.," *Cancer Immunol. Res.*, vol. 5, no. 6, pp. 480–492, 2017.

[5] K. M. Mahoney *et al.*, "A secreted PD-L1 splice variant that covalently dimerizes and mediates immunosuppression," *Cancer Immunol. Immunother.*, pp. 1–12, Dec. 2018.

[6] J. et al Orme, "ADAM10 and ADAM17 cleave PD-L1 to mediate PD-(L)1 inhibitor resistance.," *Oncoimmunology*, vol. In press, p. DOI 10.1080/2162402X.2020.1744980, 2020.

[7] Y. Romero, R. Wise, and A. Zolkiewska, "Proteolytic processing of PD-L1 by ADAM proteases in breast cancer cells," *Cancer Immunol. Immunother.*, vol. 69, no. 1, pp. 43–55, Jan. 2020.

[8] G. Chen *et al.*, "Exosomal PD-L1 contributes to immunosuppression and is associated with anti-PD-1 response," *Nature*, vol. 560, no. 7718, pp. 382–386, Aug. 2018.

[9] M. Poggio *et al.*, "Suppression of Exosomal PD-L1 Induces Systemic Anti-tumor Immunity and Memory," *Cell*, vol. 177, no. 2, pp. 414-427.e13, Apr. 2019.

[10] Y. Li *et al.*, "Serum sPD-1 and sPD-L1 as Biomarkers for Evaluating the Efficacy of Neoadjuvant Chemotherapy in Triple-Negative Breast Cancer Patients.," *Clin. Breast Cancer*, vol. 19, no. 5, pp. 326-332.e1, Oct. 2019.

[11] H. Ha *et al.*, "Soluble programmed death-ligand 1 (sPDL1) and neutrophil-to-lymphocyte ratio (NLR) predicts survival in advanced biliary tract cancer patients treated with palliative chemotherapy.," *Oncotarget*, vol. 7, no. 47, pp. 76604–76612, Nov. 2016.

[12] Y. Fan *et al.*, "Exosomal PD-L1 Retains Immunosuppressive Activity and is Associated with Gastric Cancer Prognosis.," *Ann. Surg. Oncol.*, vol. 26, no. 11, pp. 3745–3755, Oct. 2019.

[13] K. Ando *et al.*, "Plasma Levels of Soluble PD-L1 Correlate With Tumor Regression in Patients With Lung and Gastric Cancer Treated With Immune Checkpoint Inhibitors.," *Anticancer Res.*, vol. 39, no. 9, pp. 5195–5201, Sep. 2019.

[14] A. Costantini *et al.*, "Predictive role of plasmatic biomarkers in advanced non-small cell lung cancer treated by nivolumab.," *Oncoimmunology*, vol. 7, no. 8, p. e1452581, 2018.

[15] Y. Okuma *et al.*, "Soluble Programmed Cell Death Ligand 1 as a Novel Biomarker for Nivolumab Therapy for Non-Small-cell Lung Cancer.," *Clin. Lung Cancer*, vol.

19, no. 5,
pp. 410-417.e1, 2018.

[16] J. et al Orme, “Plasma exchange clears soluble PD-L1 and extracellular vesicles in vivo,”
Manuscr. Prep., 2020.

[17] X. Frigola *et al.*, “Identification of a soluble form of B7-H1 that retains immunosuppressive activity and is associated with aggressive renal cell carcinoma.,” *Clin Cancer Res. Clin Cancer Res. April*, vol. 1, no. 177, pp. 1915–1923, 2011.

[18] E. A. Eisenhauer *et al.*, “New response evaluation criteria in solid tumours: Revised RECIST guideline (version 1.1),” *Eur. J. Cancer*, vol. 45, no. 2, pp. 228–247, Jan. 2009.

[19] “Keytruda: Highlights of Presciribing Information,” 2017.

[20] “Nivolumab: Highlights of Prescribing Information,” 2018.

Appendix I ECOG Performance Status

ECOG PERFORMANCE STATUS*	
**Grade**	**ECOG**
0	Fully active, able to carry on all pre-disease performance without restriction
1	Restricted in physically strenuous activity but ambulatory and able to carry out work of a light or sedentary nature, e.g., light house work, office work
2	Ambulatory and capable of all selfcare but unable to carry out any work activities. Up and about more than 50% of waking hours
3	Capable of only limited selfcare, confined to bed or chair more than 50% of waking hours.
4	Completely disabled. Cannot carry on any selfcare. Totally confined to bed or chair.
5	Dead

*As published in Am. J. Clin. Oncol.:
Oken, M.M., Creech, R.H., Tormey, D.C., Horton, J., Davis, T.E., McFadden, E.T., Carbone, P.P.: Toxicity And Response Criteria Of The Eastern Cooperative Oncology Group. Am J Clin Oncol 5:649-655, 1982.

The ECOG Performance Status is in the public domain therefore available for public use. To duplicate the scale, please cite the reference above and credit the Eastern Cooperative Oncology Group, Robert Comis M.D., Group Chair.

From http://www.ecog.org/general/perf_stat.html

Adverse Event Form

STUDY NAME

Site Name:______________________ Pt_ID:______________________	This form is cumulative and captures adverse events of a single participant throughout the study.

Severity	Study Intervention Relationship	Action Taken Regarding Study Intervention	Outcome of AE	Expected	Serious Adverse Event (SAE)
1 = Mild<br>2 = Moderate<br>3 = Severe<br>4 = Life-Threatening	0 = Not related<br>1 = Unlikely related<br>2 = Possibly related<br>3 = Probably related<br>4 = Definitely related	0 = None<br>1 = Dose modification<br>2 = Medical Intervention<br>3 = Hospitalization<br>4 = Intervention discontinued<br>5 = Other	1 = Resolved<br>2 = Recovered with minor sequelae<br>3 = Recovered with major sequelae<br>4 = Ongoing/Continuing treatment<br>5 = Condition worsening<br>6 = Death<br>7 = Unknown	1 = Yes<br>2 = No	1 = Yes<br>2 = No<br>(if yes, complete SAE form)

At end of study only: Check this box if participant had no adverse events ☐ None

Adverse Event	Start Date	Stop Date	Severity	Relationship	Action Taken	Outcome of AE	Expected?	SAE?

Page __ of __

Adverse Event Form Version 2.0

RESEARCH PARTICIPANT CONSENT AND PRIVACY AUTHORIZATION FORM

Study Title: MC200703: Radiation Therapy, Plasma Exchange, and Immunotherapy in Melanoma

IRB#: 20-003367

Principal Investigator: Dr. Jacob Orme and Colleagues

Key Study Information

This section provides a brief summary of the study. It is important for you to understand why the research is being done and what it will involve before you decide. **Please take the time to read the entire consent form carefully and talk to a member of the research team before making your decision.** You should not sign this form if you have any questions that have not been answered.	
**It's Your Choice**	This is a research study. Being in this research study is your choice; you do not have to participate. If you decide to join, you can still stop at any time. You should only participate if you want to do so. You will not lose any services, benefits or rights you would normally have if you choose not to take part.
**Research Purpose**	The purpose of this research is to look at your blood samples and compare levels of metabolites (these are levels of vitamins, carbohydrates, proteins, etc., that are in your blood), before and after the plasma exchange (also known as "plasmapheresis" which is a way to "clean" or "flush out" your blood) that we hope will improve the effect of the standard immunotherapy treatment has on your cancer cells.<br><br>You have been asked to take part in this research because you are an adult who is scheduled to receive immunotherapy for your melanoma.

**What's Involved**	Study participation involves testing your blood before and after plasma exchange to help us see how well this procedure works to remove substances from your blood stream that affects how the immune system functions.<br><br>You will only be in the study until you are finished receiving your plasma exchange procedure(s) and the study portion is completed. However, we will follow you during your treatment and follow-up visits, for up to 2 years.
**Key Information**	The risks of drawing blood include pain, bruising, lightheadedness, and/or fainting, and rarely infection at the site of the needle.<br><br>The risks of plasma exchange are rare but may include hypotension, infection, and bleeding. You will be monitored throughout the entire procedure. Plasma exchange requires adequate vascular access. If your veins are not adequate for plasma exchange, we will place a temporary central venous access device (central line). The risks of central lines are rare but may include infection, thrombosis, and bleeding. You will be monitored throughout the procedure and the central line will be removed at the end of the third plasma exchange.<br><br>As with all research, there is a chance that confidentiality could be compromised; however, we take precautions to minimize this risk.<br>As a participant in this study, you will receive no direct benefit to your care.<br><br>Your treatment will not be changed by the results of these tests. Results of these tests will not be available to you or your providers in your medical record or elsewhere.
**Learn More**	If you are interested in learning more about this study, read the rest of this form carefully. The information in this form will help you decide if you want to participate in this research or not. A member of our research team will talk with you about taking part in this study before you sign this form. If you have questions at any time, please ask us.

Making Your Decision

Taking part in research is your decision. Take your time to decide. Feel free to discuss the study with your family, friends, and healthcare provider before you make your decision. Taking part in this study is completely voluntary and you do not have to participate.

If you decide to take part in this research study, you will sign this consent form to show that you want to take part. We will give you either a printed or electronic copy of this form to keep. A copy of this form will be put in your medical record.

For purposes of this form, Mayo Clinic refers to Mayo Clinic in Arizona, Florida and Rochester, Minnesota; Mayo Clinic Health System; and all owned and affiliated clinics, hospitals, and entities.

Contact Information

If you have questions about …	You can contact …
▪ Study tests and procedures<br>▪ Materials you receive<br>▪ Research-related appointments<br>▪ Research-related concern or complaint<br>▪ Research-related injuries or emergencies<br>▪ Withdrawing from the research study	**Principal Investigators:**<br>Dr. Jacob Orme<br>**Phone:** (507) 293-7683<br><br>Co-PI: Dr. Sean Park<br>**Phone:** (507) 422-6666<br><br>**Study Team Contact:**<br>RSTRADONCRES@mayo.edu<br><br>**Institution Name and Address:**<br>Mayo Clinic<br>Department of Medical Oncology<br>200 First Street SW<br>Rochester, MN 55905
▪ Rights of a research participant	**Mayo Clinic Institutional Review Board (IRB)**<br>**Phone:** (507) 266-4000<br>**Toll-Free:** (866) 273-4681
▪ Rights of a research participant<br>▪ Any research-related concern or complaint<br>▪ Use of your Protected Health Information<br>▪ Stopping your authorization to use your Protected Health Information<br>▪ Withdrawing from the research study	**Research Participant Advocate (RPA)**<br>**(The RPA is independent of the Study Team)**<br>**Phone:** (507) 266-9372<br>**Toll-Free:** (866) 273-4681<br><br>**E-mail:** researchparticipantadvocate@mayo.edu
▪ Billing or insurance related to this research study	**Patient Account Services**<br>**Toll-Free:** (844) 217-9591

Other Information:

A description of this clinical trial will be available on http://www.ClinicalTrials.gov, as required by U.S. Law, and is also available on http://mayo.edu/research/clinical-trials. This Web site will not include information that can identify you. At most, the Web site will include a summary of the results. You can search this Web site at any time.

Why are you being asked to take part in this research study?

You are being asked to take part in this research study because you are scheduled to receive immunotherapy for your melanoma. The goal of this study is to look at your blood samples and compare the before and aftereffects of plasmapheresis on your cancer.

Why is this research study being done?

The purpose of this research is to look at your blood samples and compare levels of metabolites (these are levels of vitamins, carbohydrates, proteins, etc., that are in your blood), before and after the plasma exchange (also known as "plasmapheresis" which is a way to "clean" or "flush out" your blood) that we hope will improve the effect of the standard immunotherapy treatment has on your cancer cells.

Information you should know

Who is Funding the Study?

Funding for this research comes from the Department of Medical Oncology and the National Institute of Health (NIH).

Information Regarding Conflict of Interest:

Your healthcare provider may be referring you to this research study. If your healthcare provider is also an investigator on this study, there is the chance that his or her responsibilities for the study could influence his or her recommendation for your participation.

If you prefer, your healthcare provider will be happy to refer you to another investigator on the research study team for you to decide if you want to participate in the study and to see you for the research study activities while you are in the study.

How long will you be in this research study?

You will be in this study for up to 2 years.

What will happen to you while you are in this research study?

If you agree to be in the study, you will be asked to participate in the following:

- Procedures such as: Vascular assessment, lab draws including research bloods, pregnancy test, Temporary line placement (in patients without adequate venous access)
- Therapeutic Plasma Exchange Routine Imaging
- Temporary Central line removal (in patients with line)

Some of these exams, tests or procedures are part of regular clinical care and may be done even if you do not join the study. If you have had some of them recently, they may not need to be repeated. This will be up to the Principal Investigator.

Follow up visits that include the review of any side effects (good/bad), will be completed as per the Radiation Oncology clinician's discretion and will be assessed in Radiation Oncology by the Radiation Oncologist.

Screening Visit (≤30 days prior to registration):

- Pregnancy test (for women of childbearing potential)
- **#1 Research blood draw ** to determine eligibility (Pre-RT)**

If the levels of the research blood draw are **not** within the required limits for the study, you will not be able to participate in the research portion of your treatment plan. However, you will continue on with the treatment plan without being in this study.

If your blood test **is** within the required limits for the study you will continue on with line placement (if needed), the research blood draws, and the Therapeutic Plasma Exchange (TPE) as planned with your treating doctor.

Radiation Therapy (1 – 5 days of RT):

- Treatment will be delivered daily except on weekends and holidays, per the discretion of the radiation oncologist
- Treatment may not occur on consecutive days
- **#2 Research blood draw** (after the last RT day, just before starting the TPE procedure)

Therapeutic plasma exchange (TPE) procedure:

- After you finish the RT, you will start of the Therapeutic Plasma Exchange (TPE) in three sessions over a total of three (3) days in a row. One session per day.

Therapeutic plasma exchange (TPE) will take place in our outpatient Apheresis Clinic and take 1-2 hours for each session. In TPE, two peripheral IVs will be placed (one in each arm) or a temporary central access line will be placed for the procedure. Blood will be drawn out of IV or line port, filtered, and returned to the other IV or line port with albumin replacement fluid. The purpose of TPE is to measure the removal of substances from the blood we think may be stopping immunotherapy from working in some patients. Blood samples will be collected during TPE and so additional needle sticks will be needed. Blood product transfusion is *not* planned unless needed to prevent bleeding. If bleeding occurs or appears likely, human plasma will be given to prevent or stop this bleeding.

After the third and final Therapeutic Plasma Exchange procedure:

- **#3 Research blood draw** to be performed as part of TPE (no new needle stick) – Done after your last TPE procedure
- Removal of central line (if you have one) after blood draw
- Within 5 – 7 days after completion of the TPE, you will receive and start your planned immunotherapy (pembrolizumab or nivolumab) as scheduled by your oncologist.

2-3 weeks after Radiation Therapy and after the Therapeutic Plasma Exchange procedures:

- Toxicity assessment
- **#4 Research blood draw (**follow-up at standard clinical appointment prior to your next round of immunotherapy**)**

You will then continue with your therapy as planned by your oncologists.
Follow up as clinically indicated:

- Toxicity Assessment

Progression:

- **#5 Research blood draw** - If your disease progresses or comes back, we will collect one final research blood draw.

Overall, you will have five (5) blood samples, 36 ml at each collection, collected to help determine how well the plasmapheresis procedure removes substances from your blood stream that affect how the immune system functions in response to immunotherapy against your melanoma. We would also like to obtain waste product from the plasmapheresis procedure to measure this metabolite and treat other cells to see its effects.

Tests done only for research purposes are not meant to provide clinical information or help care for you. The results are only important for research. Therefore, the results of tests done with your information and samples will not be provided to you. In the rare event that a finding might affect the health of you or your family, we will contact you and you can choose whether to receive or refuse the information. If you decide to follow up and further medical testing or care is needed, the costs will be billed to you or your insurance.

What are the possible risks or discomforts from being in this research study?

<u>Blood Draw Risks</u>
The risks of drawing blood include pain, bruising, lightheadedness, and/or fainting, and rarely infection at the site of the needle.

<u>Confidentiality Risks</u>
As with all research, there is a chance that confidentiality could be compromised; however, we take precautions to minimize this risk.

<u>Therapeutic Plasma Exchange (TPE) Risks</u>:
Complications with (TPE) that occasionally occur:

- Seepage of fluids at the needle site
- Bleeding
- Bruising (hematoma)
- Mild inflammation
- Infection
- Chills or feeling cold (can occur due to blood being removed temporarily)
- Allergic reactions caused by albumin and other procedure fluids used
- Hypocalcemia (low calcium levels)
- Hypokalemia (low potassium levels)
- Tingling in the lips, hands, and feet
- Feelings of anxiousness
- Feeling lightheaded for a short time due to the removal of blood.

- Embolism (air entering the veins)
- Fever
- Arrhythmias (abnormal heart rhythm)
- Drop in blood pressure.

We hope that the use of TPE may improve the cancer's response to the immunotherapy. Similarly, TPE may increase autoimmune side effects such as autoimmune colitis (inflammatory bowel disease). You will be monitored carefully throughout the procedure and every effort will be made to make you comfortable and to prevent and treat these complications. Treatments to help with side effects may include additional fluids, blood products, anti-inflammatory medications, and antibiotics. Should any adverse effect be noted, plasma exchange will be stopped, and safety will be reassessed.

Central Line Risks (only used in the event of inadequate venous access):
Complications with central lines that occasionally occur:

- Seepage of fluids at the line placement site
- Bleeding
- Bruising (hematoma)
- Thrombosis (clotting)
- Mild inflammation
- Infection
- Chills or feeling cold (can occur due to blood being removed temporarily)

In people without adequate peripheral veins, the use of a central line will allow therapeutic plasma exchange to take place. This line will be placed 48-72 hours before the plasma exchange to reduce the risk of bleeding. It will be removed immediately after the last session of TPE to reduce the risk of infection and thrombosis. Should any adverse effect be noted, line placement will be stopped and safety will be reassessed.

Unknown Risks:
During the course of procedure, unforeseen conditions may be revealed or occur.

Birth Control (Male & Female)
If you are sexually active and able to become pregnant or able to father a child, you must use birth control for the entire study and you must agree to use one of the birth control methods listed below, prior to starting any radiation therapy and after:

- Hormonal methods, such as birth control pills, patches, injections, vaginal ring, or implants
- Barrier methods (such as a condom or diaphragm) used with a spermicide (a foam, cream, or gel that kills sperm)

- Intrauterine device (IUD)
- Abstinence (no sex)

You must use birth control for the entire study, up to 2 years. You must also use birth control for at least 5 months after your last dose of Pembrolizumab or Nivolumab.

If you are a female of childbearing potential, you must have a negative pregnancy test in order to participate in this study unless you cannot become pregnant.
Standard of Care Risks
Your doctor will discuss the risks of tests and procedures that are part of your standard clinical care including imaging and blood draws.

Are there reasons you might leave this research study early?

You may decide to stop at any time. You should tell the Principal Investigator if you decide to stop and you will be advised whether any additional tests may need to be done for your safety.

In addition, the Principal Investigator or Mayo Clinic may stop you from taking part in this study at any time:

- If it is in your best interest,
- If you don't follow the study procedures,
- If the study is stopped.

If you leave this research study early, or are withdrawn from the study, no more information about you will be collected; however, information already collected about you in the study may continue to be used.

We will tell you about any new information that may affect your willingness to stay in the research study.

What if you are injured from your participation in this research study?

Where to get help:

If you think you have suffered a research-related injury, you should promptly notify the Principal Investigator listed in the Contact Information at the beginning of this form. Mayo Clinic will

offer care for research-related injuries, including first aid, emergency treatment and follow-up care as needed.

Who will pay for the treatment of research related injuries:

Care for such research-related injuries will be billed in the ordinary manner, to you or your insurance. Treatment costs for research-related injuries not covered by your insurance will be paid by Mayo Clinic.

What are the possible benefits from being in this research study?

This study may not make your health better. However, with your help, researchers will better understand the side effects from each treatment and possibly lessen those side effects for future treatments. Others with melanoma may benefit in the future from what we learn in this research study.

What alternative do you have if you choose not to participate in this research study?

You don't have to be in this study to receive treatment for your condition. Your other choices may include;

- Receiving treatment or care for your cancer without being in the study
- Taking part in another study
- Getting no treatment

Talk to the Principal Investigator or your doctor if you have any questions about any of these treatments or procedures.

What tests or procedures will you need to pay for if you take part in this research study?

You won't need to pay for tests and procedures which are done just for this research study. These tests and procedures are:

- Research Blood Draws

  - sPD-L1 – Screening
  - Research bloods (up to 5 time-points)
- Central line placement (if needed)
- Therapeutic Plasma Exchange (TPE)
  - Albumin (used with TPE procedure)

However, you and/or your insurance will need to pay for all other tests and procedures that you would have as part of your clinical care.

These tests and procedures are:
- Routine Imaging
  - CT, MRI, etc., at the discretion of the treating physician
  - Pregnancy testing (if a woman of child-bearing potential)
  - Toxicity Assessments
- Pembrolizumab or nivolumab (Immunotherapy – Per physician preference)
- Radiation Therapy

You will also be responsible for any co-payments and deductibles.

If you have billing or insurance questions call Patient Account Services at the telephone number provided in the Contact Information section of this form.

Will you be paid for taking part in this research study?

There is a very small chance that some commercial value may result from the use of your sample. This could include new products like a drug or a test to diagnose a disease. If that happens, you will not be offered a share in any profits.

You won't be paid for taking part in this study.

Will your information or samples be used for future research?

We would like to keep your information and samples for future research. You can still take part in this current study even if you don't want your information or samples used for future research.

Researchers at Mayo Clinic who aren't involved with this study may ask to use your information and/or samples for future research. Researchers at other institutions may also ask for a part of your information and/or samples for future studies. Unless you indicate otherwise, the future research may be on any topic. No direct benefits to you are expected from the future research. Your information and/or samples will only be shared consistent with your consent, and with all applicable laws and regulations.

If you approve release of your information and/or samples by checking 'yes' below, Mayo may send the information and/or samples to researchers who request them, but Mayo will not send your name, address, phone number, social security number, or any other identifying information with the information and/or samples. Your information and/or samples may be sent with a code, and only the researchers for this study at Mayo Clinic would be able to link the code to you.

Please read the following statements and mark your choices:

1. I permit my information and samples to be stored and used in future research of cancer at Mayo Clinic:

☐ Yes ☐ No Please initial here: ________Date: ________

2. I permit my information and samples to be stored and used in future research at Mayo Clinic to learn about, prevent, or treat any other health problems:

☐ Yes ☐ No Please initial here: ________Date: ________

3. I permit Mayo Clinic to give my information and samples to researchers at other institutions:

☐ Yes ☐ No Please initial here: ________Date: ________

You may withdraw your consent for future use of your information and/or samples at any time, by writing to the Principal Investigator at the address provided in the "Contact Information" section of this consent form.

Your information and/or samples would be removed from any repository where they are stored, if possible. Information and/or samples already distributed for research use will not be retrieved. When you die, your sample will be considered a gift to Mayo Clinic. That means that Mayo Clinic can use it for research forever. Since your sample has your genetic information in it, your family may want access to it after you die. They can use that information for many things, such as learning if you had a genetic disease or if you were related to someone.

Read the following statement and mark your choice:

I permit Mayo Clinic to give my family access to my sample after I die:

☐ Yes ☐ No Please initial here: ________ Date: ________

How will your privacy and the confidentiality of your records be protected?

Blood and waste samples are stored at Mayo Clinic in secured freezers. Research data is stored in files on computers which are password protected. If the results of the research are made public, information that identifies you will not be used.

Mayo Clinic is committed to protecting the confidentiality of information obtained about you in connection with this research study. Samples will be given research-specific identifiers that are separated from clinically identifiable information. These data will be stored in files on computers which are password protected.

During this research, information about your health will be collected. Under Federal law called the Privacy Rule, health information is private. However, there are exceptions to this rule, and you should know who may be able to see, use and share your health information for research and why they may need to do so. Information about you and your health cannot be used in this research study without your written permission. If you sign this form, it will provide that permission (or "authorization") to Mayo Clinic.

Your health information may be collected from:

- Past, present and future medical records.
- Research procedures, including research office visits, tests, interviews and questionnaires.

Your health information will be used and/or given to others to:

- Do the research.
- Report the results.
- See if the research was conducted following the approved study plan, and applicable rules and regulations.

Your health information may be used and shared with:

- Mayo Clinic research staff involved in this study.
- Other Mayo Clinic staff involved in your clinical care.

- The Mayo Clinic Institutional Review Board that oversees the research.
- Federal and State agencies (such as the Food and Drug Administration, the Department of Health and Human Services, the National Institutes of Health and other United States agencies) or government agencies in other countries that oversee or review research.
- A group that oversees the data (study information) and safety of this research.

How your information may be shared with others:

While taking part in this study, you will be assigned a code that is unique to you, but does not include information that directly identifies you. This code will be used if your study information is sent outside of Mayo Clinic. The groups or individuals who receive your coded information will use it only for the purposes described in this consent form.

If the results of this study are made public (for example, through scientific meetings, reports or media), information that identifies you will not be used.

In addition, individuals involved in study oversight and not employed by Mayo Clinic may be allowed to review your health information included in past, present, and future medical and/or research records. This review may be done on-site at Mayo Clinic or remotely (from an off-site location). These records contain information that directly identifies you. However, the individuals will not be allowed to record, print, or copy (using paper, digital, photographic or other methods), or remove your identifying information from Mayo Clinic.

Is your health information protected after it has been shared with others?

Mayo Clinic asks anyone who receives your health information from us to protect your privacy; however, once your information is shared outside Mayo Clinic, we cannot promise that it will remain private and it may no longer be protected by the Privacy Rule.

Your Rights and Permissions

Participation in this study is completely voluntary. You have the right not to participate at all. Even if you decide to be part of the study now, you may change your mind and stop at any time. You do not have to sign this form, but if you do not, you cannot take part in this research study.

Deciding not to participate or choosing to leave the study will not result in any penalty. Saying ‘no’ will not harm your relationship with your own doctors or with Mayo Clinic.

If you cancel your permission for Mayo Clinic to use or share your health information, your participation in this study will end and no more information about you will be collected; however, information already collected about you in the study may continue to be used.

You can cancel your permission for Mayo Clinic to use or share your health information at any time by sending a letter to the address below:

Mayo Clinic
Office for Human Research Protection
ATTN: Notice of Revocation of Authorization
201 Building 4-60
200 1st Street SW
Rochester, MN 55905

Alternatively, you may cancel your permission by emailing the Mayo Clinic Research Participant Advocate at: researchparticipantadvocate@mayo.edu.

Please be sure to include in your letter or email:

- The name of the Principal Investigator,
- The study IRB number and /or study name, and
- Your contact information.

Your permission for Mayo Clinic to use and share your health information lasts forever, unless you cancel it.

Enrollment and Permission Signatures

Your signature documents your permission to take part in this research.

/ /	: AM/PM

Printed Name Date Time

____
Signature

Person Obtaining Consent

- I have explained the research study to the participant.
- I have answered all questions about this research study to the best of my ability.

/ /	: AM/PM

Printed Name Date Time

____
Signature
