# Supplementary material for: Plasma exchange and radiation resensitize immunotherapy-refractory melanoma: a phase I trial
Source: Nat Commun. 2025 Mar 13;16:2507. doi: 10.1038/s41467-025-57865-9 (PMC11906856; doi:10.1038/s41467-025-57865-9)
Supplement: Supplementary file 2 — Reporting Summary [file 41467_2025_57865_MOESM2_ESM.pdf]

nature portfolio

Corresponding author(s): Jacob Orme, MD PhD

Last updated by author(s): 02/12/2025

Reporting Summary

Nature Portfolio wishes to improve the reproducibility of the work that we publish. This form provides structure for consistency and transparency in reporting. For further information on Nature Portfolio policies, see our Editorial Policies and the Editorial Policy Checklist.

Statistics

For all statistical analyses, confirm that the following items are present in the figure legend, table legend, main text, or Methods section.

n/a	Confirmed	
☐	☒	The exact sample size (n) for each experimental group/condition, given as a discrete number and unit of measurement
☐	☒	A statement on whether measurements were taken from distinct samples or whether the same sample was measured repeatedly
☐	☒	The statistical test(s) used AND whether they are one- or two-sided<br>*Only common tests should be described solely by name; describe more complex techniques in the Methods section.*
☐	☒	A description of all covariates tested
☐	☒	A description of any assumptions or corrections, such as tests of normality and adjustment for multiple comparisons
☐	☒	A full description of the statistical parameters including central tendency (e.g. means) or other basic estimates (e.g. regression coefficient) AND variation (e.g. standard deviation) or associated estimates of uncertainty (e.g. confidence intervals)
☐	☒	For null hypothesis testing, the test statistic (e.g. F, t, r) with confidence intervals, effect sizes, degrees of freedom and P value noted<br>*Give P values as exact values whenever suitable.*
☒	☐	For Bayesian analysis, information on the choice of priors and Markov chain Monte Carlo settings
☒	☐	For hierarchical and complex designs, identification of the appropriate level for tests and full reporting of outcomes
☒	☐	Estimates of effect sizes (e.g. Cohen's d, Pearson's r), indicating how they were calculated

Our web collection on statistics for biologists contains articles on many of the points above.

Software and code

Policy information about availability of computer code

Data collection	Data was collected prospectively in a Meditata Rave Electronic Data Capture (EDC) System (2021).
Data analysis	Data analysis was performed using R! computing software (v4.4.1).

For manuscripts utilizing custom algorithms or software that are central to the research but not yet described in published literature, software must be made available to editors and reviewers. We strongly encourage code deposition in a community repository (e.g. GitHub). See the Nature Portfolio guidelines for submitting code & software for further information.

Data

Policy information about availability of data

All manuscripts must include a data availability statement. This statement should provide the following information, where applicable:

- Accession codes, unique identifiers, or web links for publicly available datasets
- A description of any restrictions on data availability
- For clinical datasets or third party data, please ensure that the statement adheres to our policy

All raw data, including redacted individual patient data, and a data dictionary defining each field is available in Supplementary Data File 1 (https://doi.org/10.6084/m9.figshare.27317040). Data underlying each figure are separately available in Source Data File 1. Study protocol and informed consent form are available in the supplementary information.

Research involving human participants, their data, or biological material

Policy information about studies with human participants or human data. See also policy information about sex, gender (identity/presentation), and sexual orientation and race, ethnicity and racism.

Reporting on sex and gender	Consent for sex reporting was obtained and is reported in the manuscript. To comply with de-identification rules, individual sex is not reported in Data File 1. This early trial is not sufficiently powered to draw conclusions based on sex differences.
Reporting on race, ethnicity, or other socially relevant groupings	Race/ethnicity was not reported in our dataset.
Population characteristics	Consent was obtained and we provide age, diagnosis, and treatment information for all patients in the study.
Recruitment	Patients were recruited according to the attached protocol. In brief, patients with metastatic melanoma progressing despite ICI treatment were approached at a single academic center between December 2020 and February 2023. Patients were screened for sPD-L1 levels using a sandwich enzyme-linked immunosorbent assay (ELISA) kit (see Biomarker Assays). Patients with sPD-L1 levels ≥1.7 ng/mL were considered positive and eligible for the study. Patients were not compensated for study participation.
Ethics oversight	We conducted the ReCIPE-M1 (Rescuing Cancer Immunotherapy with Plasma Exchange in Melanoma) study under a protocol approved by an institutional review board and registered at ClinicalTrials.gov (NCT04581382). Study design and conduct complied with all relevant regulations regarding the use of human study participants and was conducted in accordance with the criteria set by the Declaration of Helsinki.

Note that full information on the approval of the study protocol must also be provided in the manuscript.

Field-specific reporting

Please select the one below that is the best fit for your research. If you are not sure, read the appropriate sections before making your selection.

- [x] Life sciences
- [ ] Behavioural & social sciences
- [ ] Ecological, evolutionary & environmental sciences

For a reference copy of the document with all sections, see nature.com/documents/nr-reporting-summary-flat.pdf

Life sciences study design

All studies must disclose on these points even when the disclosure is negative.

Sample size	The primary endpoint of this trial was to assess the feasibility and kinetics for this treatment approach. Feasibility was assessed by being able to complete the study accrual in a reasonable time period (this was met). Kinetics of sPD-L1 production in melanoma were assessed in an exploratory fashion by assessing the change in the sPD-L1 levels between different time points of interest. Since this feasibility study was small and exploratory, we estimated 17 evaluable patients as sufficient for secondary efficacy with an expected response rate of 10-20%. Additional comparator substances or potentially pathogenic tumor substances were measured for comparison.
Data exclusions	No data was excluded from analysis.
Replication	All peripheral blood studies (Olink proteomics and PBMC flow cytometry) were performed in triplicate by a blinded technician.
Randomization	Patients were not randomized in this study. However, samples were randomized across plates for both flow cytometry and proteomic studies.
Blinding	All peripheral blood studies (Olink proteomics and PBMC flow cytometry) were performed in triplicate by a blinded technician.

Reporting for specific materials, systems and methods

We require information from authors about some types of materials, experimental systems and methods used in many studies. Here, indicate whether each material, system or method listed is relevant to your study. If you are not sure if a list item applies to your research, read the appropriate section before selecting a response.

Materials & experimental systems

n/a	Involved in the study	
	x	Antibodies
x		Eukaryotic cell lines
x		Palaeontology and archaeology
x		Animals and other organisms
	x	Clinical data
x		Dual use research of concern
x		Plants

Methods

n/a	Involved in the study	
x		ChIP-seq
	x	Flow cytometry
x		MRI-based neuroimaging

Antibodies

Antibodies used

Antibody Vendor Clone Catalog no.
CD3 BV750 Biolegend SK7 344846
CD8 PE/Cy7 BD Pharmingen RPA-T8 557746
CD11a APC Biolegend HI111 301212
PD-1 BV605 Biolegend EH12.2H7 329924
CX3CR1 APC/Cy7 Biolegend 2A9-1 341616
Granzyme B Percp Novus Biological CLB-GB11 NBP-1-50071PCP
Bim PE Cell Signaling C34C5 12186S
Ki-67 BV421 BD Horizon B56 562899
NKG7 In-house, Fusion Antibodies 8H3/8K3 NA
CD3 PE/Cy7 Biolegend HIT3a 300316
CD4 FITC Biolegend SK3 344604
CD25 BV421 Biolegend 2A3 302630
CD8 PE Biolegend SK1 344705
CD45RA PerCP Biolegend HI100 304156
CCR7 BV785 Biolegend G043H7 353230
FoxP3 Alexa Fluor 647 BD Pharmingen 236A/E7 561184
Lin1(CD3, CD19, CD20, CD56) APC Biolegend UCHT1; HIB19; 2H7; 5.1H11; 363601
CD14 BV421 BD Horizon MφP9 563743
CD16 PerCP Biolegend 3G8 302030
HLA-DR APC-H7 BD Pharmingen G46-6 561358
CD11c FITC BD Pharmingen B-ly6 561355
CD123 PE-Cy BD Pharmingen 7G3 560826
CD1c PE BD Pharmingen F10/21A3 564900
CD141 BV650 BD Horizon 1A4 569392
CCR2 BUV661 BD OptiBuild LS132.1D9 750472
CD86 PE/Dazzle™ 594 Biolegend BU63 374218
PD-1 BV785 Biolegend EH12.2H7 329930
PD-L1 BUV395 BD OptiBuild MIH1 740320

Validation

As outlined in Supplemental Tables 2 and 3 and in the methods, each antibody was purchased new from the manufacturer and subjected to quality control on known samples prior to testing.

Clinical data

Policy information about clinical studies
All manuscripts should comply with the ICMJE guidelines for publication of clinical research and a completed CONSORT checklist must be included with all submissions.

Clinical trial registration	NCT04581382
Study protocol	Uploaded with the submission
Data collection	2021-2024
Outcomes	Outcomes were prespecified in the attached study protocol and approved prior to study start.

Plants

Seed stocks

Report on the source of all seed stocks or other plant material used. If applicable, state the seed stock centre and catalogue number. If plant specimens were collected from the field, describe the collection location, date and sampling procedures.

Novel plant genotypes

Describe the methods by which all novel plant genotypes were produced. This includes those generated by transgenic approaches, gene editing, chemical/radiation-based mutagenesis and hybridization. For transgenic lines, describe the transformation method, the number of independent lines analyzed and the generation upon which experiments were performed. For gene-edited lines, describe the editor used, the endogenous sequence targeted for editing, the targeting guide RNA sequence (if applicable) and how the editor was applied.

Authentication

Describe any authentication procedures for each seed stock used or novel genotype generated. Describe any experiments used to assess the effect of a mutation and, where applicable, how potential secondary effects (e.g. second site T-DNA insertions, mosiacism, off-target gene editing) were examined.

Flow Cytometry

Plots

Confirm that:

☒ The axis labels state the marker and fluorochrome used (e.g. CD4-FITC).

☒ The axis scales are clearly visible. Include numbers along axes only for bottom left plot of group (a 'group' is an analysis of identical markers).

☒ All plots are contour plots with outliers or pseudocolor plots.

☒ A numerical value for number of cells or percentage (with statistics) is provided.

Methodology

Sample preparation

Samples were obtained from Ficoll exclusion and resuspended as a single cell suspension in cryopreservative before resuspension and staining for flow cytometry in bulk.

Instrument

Cytek Aurora (Cytek Biosciences)

Software

FlowJo 10.10.0 (Tree Star)

Cell population abundance

No cell sorting was performed for post-sort manipulation or analysis. Cell populations as specified are listed in both the patient data file and supplemental tables.

Gating strategy

FSC-A and SSCA gates were performed for lymphocytes as outlined in Supplemental Figure 7. Further live cells (between 93 and 100%) were subclassified by viability Zombie UV-negative. All other stains were marked as positive and negative per isotype controls (see Supplemental Figures 6 and 7).

☒ Tick this box to confirm that a figure exemplifying the gating strategy is provided in the Supplementary Information.
