# Supplementary material for: Plasma exchange and radiation resensitize immunotherapy-refractory melanoma: a phase I trial
Source: Nat Commun. 2025 Mar 13;16:2507. doi: 10.1038/s41467-025-57865-9 (PMC11906856; doi:10.1038/s41467-025-57865-9)
Supplement: Supplementary file 3 — Description of Additional Supplementary Files [file 41467_2025_57865_MOESM3_ESM.pdf]

Nature Communications: Supplementary Data 1 Legend

Title: Plasma exchange and radiation resensitize immunotherapy-refractory melanoma: a phase I trial.

Tab: Clinical

Clinical data underlying the manuscript are shown. Individual patient data are given as rows. Columns indicate the following:

PtID: De-identified patient ID corresponding with PtID in Radiation, PBMC, and Olink data (see below).

BRAF_status: Wild-type signifies no BRAF mutation; other values signify the particular amino acid change in standard format.

TPE_total: The number of daily therapeutic plasma exchange (TPE) sessions the patient underwent

Best_response: The best recorded radiographic response the patient experienced on the trial intervention

Immunotherapy: The immunotherapy regimen received by the patient on the trial intervention

Prior_exposure: Whether (Y) or not (N) the patient had previously received the intervention listed under “Immunotherapy”

Prior_therapies_n and list: The number and names of previously-received therapies are listed for each patient.

Prior_ICI_best_response: The best response the patient had previously experienced on imunotherapy.

Prior_resistance_type: The type of resistance (primary versus secondary) to ICI therapy.

OS_days: The number of days to last OS follow-up

OS_text: The patient’s status at OS follow-up (Alive versus Death)

OS_status: The patient’s status (Alive = 0, Death = 1)

sPDL1_Screening: The measured level of soluble PD-L1 in ng/mL at screening (baseline).

sPDL1_preTPE: The measured level of soluble PD-L1 in ng/mL after SBRT and prior to TPE (pre-TPE).

sPDL1_postTPE: The measured level of soluble PD-L1 in ng/mL after TPE and before ICI re-challenge (post-TPE)

sPDL1_nextcycle: The measured level of soluble PD-L1 in ng/mL before the second cycle of ICI re-challenge (ICI2)

pfs_days: The number of days to last PFS follow-up

pfs_status: The patient’s status at PFS follow-up (Progression-free = 0, Progression = 1)

Tab: Radiation

Radiation administration data are shown. Individual lesion data are given as rows. Columns indicate the following:

- PtID: De-identified patient ID corresponding with PtID in Clinical, PBMC, and Olink data.
- Radiated_lesion_sites: A list of sites that were radiated in each patient
- Radiation_dose_Gy: The dose of radiation per fraction given to each site in Gray units
- Radiation_fractions: The number of fractions given to each site
- Total_lesion_dose: The total dose of radiation given to each site in Gray units
- Total_patient_dose: The total dose of radiation given to the patient
- Radiated_lesions_n: Number of radiated lesions for each patient
- Unirradiated_lesions_n: Number of unirradiated lesions for each patient
- pct_irradiated: Percent of lesions irradiated for each patient
- Radiated_lesion_types: Extent of radiation to known sites of disease

Tab: PBMC

Peripheral blood mononuclear cell population data are shown. Individual patient and timepoint data are given as rows. Columns indicate the following:

Study ID: De-identified patient ID corresponding with PtID in Clinical, Radiation, and Olink data.

Timepoint: The time of the sample for each patient

Cell types (columns D-AH): The percent of all cells staining positive for markers of the given cell type.

Tab: Olink

Peripheral blood protein expression data are shown. Individual patient and timepoint data are given as columns E-CP. Each analyte is outlined in columns A-D as follows:

UniProt: UniProt identifier of the specified analyte

Assay: Abbreviation of the specified analyte

Gene_Description: Full name of the specified analyte

Panel: The Olink proprietary panel set in which each target analyte is categorized.
